# *Metabacillus dongyingensis* sp. nov. Is Represented by the Plant Growth-Promoting Bacterium BY2G20 Isolated from Saline-Alkaline Soil and Enhances the Growth of *Zea mays* L. under Salt Stress

**DOI:** 10.1128/msystems.01426-21

**Published:** 2022-03-01

**Authors:** Zhiqiu Yin, Xin Wang, Yujie Hu, Jikun Zhang, Hui Li, Yanru Cui, Dongying Zhao, Xusheng Dong, Xiaohang Zhang, Kai Liu, Binghai Du, Yanqin Ding, Chengqiang Wang

**Affiliations:** a College of Life Sciences and Shandong Engineering Research Center of Plant-Microbial Restoration for Saline-Alkali Land and State Key Laboratory of Crop Biology, Shandong Agricultural University, Tai’an, China; b National Engineering Laboratory for Efficient Utilization of Soil and Fertilizer Resources, College of Resources and Environment, Shandong Agricultural University, Tai’an, China; c Ruminant Nutrition and Physiology Laboratory, College of Animal Science and Technology, Shandong Agricultural University, Tai’an, China; d Novo Nordisk (China) Pharmaceuticals Co. Ltd., Tianjin, China; LanzaTech

**Keywords:** comparative genomics, *Metabacillus dongyingensis*, PGPR, plant growth promotion, saline-alkaline tolerance, bacillus dongying

## Abstract

A novel plant growth-promoting rhizobacterium (PGPR), which was designated strain BY2G20, was isolated from saline-alkaline soil in Dongying, China. Strain BY2G20 can grow at a NaCl range from 0 to 7% and a pH range from 7 to 9 and can prevent the growth of the phytopathogen Ralstonia solanacearum. Based on its phenotypic and genomic characteristics and phylogenetic analysis, strain BY2G20 represents a novel species of the genus *Metabacillus*, for which the name *Metabacillus dongyingensis* sp. nov. is proposed. Comparative genomic analysis of strain BY2G20 with its closely related species exhibited a high level of evolutionary plasticity derived by horizontal gene transfer, which facilitated adaptative evolution. Different evolutionary constraints have operated on the diverse functions of BY2G20, with the gene adapted to saline-alkaline ecosystems experiencing functional constraints. We determined the genetic properties of saline-alkaline tolerance and plant growth promotion, such as cation-proton antiporters, cation transporters, osmoprotectant synthesis and transport, H^+^-transporting F_1_F_0_-ATPase, indole-3-acetic acid production, and secondary metabolite synthesis. We also evaluated the effects of strain BY2G20 on the growth of Zea mays L. (maize) under salt stress. The physiological parameters of maize such as plant height, stem diameter, dry biomass, and fresh biomass were significantly higher after inoculating strain BY2G20 under salt stress, indicating that inoculation with BY2G20 enhanced the growth of maize in saline areas. This study demonstrates that *M. dongyingensis* sp. nov. BY2G20 is a potential candidate for organic agriculture biofertilizers in saline-alkaline areas.

**IMPORTANCE** Plant growth and yield are adversely affected by soil salinity. PGPRs can promote plant growth and enhance plant tolerance to salt stress. In this study, a saline-alkaline tolerant PGPR strain BY2G20 was isolated from the rhizosphere of *Ulmus pumila* in Dongying, China. Strain BY2G20 represents a novel species within the genus *Metabacillus* based on phenotypic, genomic, and phylogenetic analysis. Genomic components have undergone different functional constraints, and the disparity in the evolutionary rate may be associated with the adaptation to a specific niche. Genomic analysis revealed numerous adaptive features of strain BY2G20 to a saline-alkaline environment and rhizosphere, especially genes related to salt tolerance, pH adaptability, and plant growth promotion. Our work also exhibited that inoculation of strain BY2G20 enhanced the growth of maize under salt stress. This study demonstrates that PGPRs play an important role in stimulating salt tolerance in plants and can be used as biofertilizers to enhance the growth of crops in saline-alkaline areas.

## INTRODUCTION

The growth, development, and productivity of crop plants are adversely affected by various environmental stresses such as drought, flooding, salinity, heat, cold, and heavy metals (1). Salinity is a major threat to agricultural productivity. It inhibits the growth and development of plants by affecting many physiological, biochemical, and metabolic processes (2). Salinity may cause nutrient deficiencies or imbalances via the competition of Na^+^ and Cl^−^ with nutrients such as K^+^, Ca^2+^, and NO^−^ (3). To improve the efficient use of saline soils for agricultural production, the utilization of plant growth-promoting rhizobacteria (PGPRs) could be an important strategy to improve plant cultivation in saline soils.

PGPRs are known to promote plant growth through the production of indole-3-acetic acid (IAA), siderophores, hydrogen cyanate and nitrogenase, and phosphate solubilization. They also possess heavy metal detoxifying activity, enhance salinity tolerance, and are effective in the biological control of plant pathogens, and so forth (2, 4–6). The genus *Bacillus* comprises typical species of PGPRs that are applied as biofertilizers and competitors of plant pathogens to enhance plant growth, for example, by promoting seedling emergence, enhancing plant biomass, and controlling disease (7). Furthermore, members of the genus *Bacillus* are widely distributed in the air, soil, and water, and have a wide range of physiological and biochemical characteristics from psychrophilic to thermophilic, acidophilic to alkaliphilic, and halophilic (8). Several alkaliphilic, alkalitolerant, or halophilic species of *Bacillus* have been isolated from different saline-alkaline habitats. The potential of *Bacillus* isolates for commercial development is enhanced by their high growth rate and adaptation to adverse ecological niches (9). Genome sequencing and comparative genomic analysis of novel *Bacillus* isolates will help us understand the molecular mechanisms and adaptive evolution of the beneficial activity of PGPR, as well as promote the development of PGPR-assisted phytoremediation technology (7, 10).

Here, we report a novel PGPR, BY2G20, which was isolated from the saline-alkaline soil of Dongying, Shandong Province, China. We sequenced the genome of strain BY2G20 and compared it to the genomes of closely related species. A polyphasic approach that integrated phylogenetic, genomic, and phenotypic analysis was used to characterize strain BY2G20; a representative of the novel species “*Metabacillus dongyingensis*.” The type strain is BY2G20^T^ (CGMCC 22867^T^). Comparative genomic analysis will help elucidate the evolution of this species. Systemic analysis based on the BY2G20 genome will provide a genetic basis for plant growth-promoting characteristics and adaptation to a high saline-alkaline environment. The effect of inoculation of strain BY2G20 on plant growth under salt stress was also evaluated.

## RESULTS AND DISCUSSION

### Collection and phenotypic characteristics of strain BY2G20.

Strain BY2G20 was isolated and purified from a saline-alkaline soil sample collected from the rhizosphere of white elm in Dongying, China (geographic coordinates 118.774959 and 37.559671), using the 10-fold dilution method on lysogeny broth (LB) medium.

**FIG 1:**
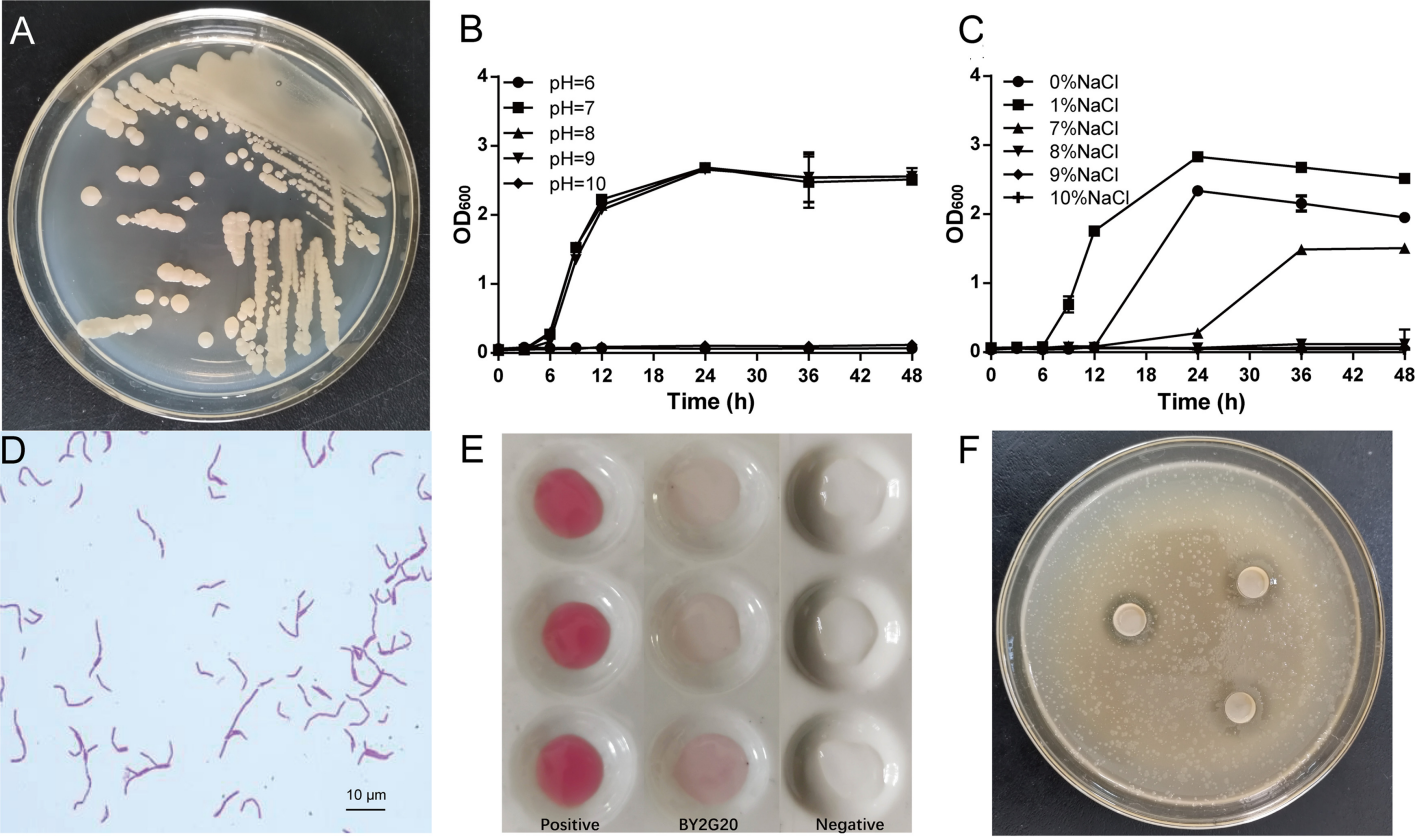
Phenotypic characteristics of strain BY2G20. (A) The morphological characteristics of the colony. (B and C) Growth curves of strain BY2G20 on different concentrations of NaCl (B) and different pH values (C). (D) Single colonies of strain BY2G20 were inoculated in LB liquid medium for 12 h at 37°C and then cultured in fresh LB liquid medium with different concentrations of NaCl and different pH values. The morphological characteristics of a single cell. The newly cultivated strain BY2G20 was placed on LB solid plate and incubated for 1 day at 37°C. (E) IAA secretion detection. A single colony of strain BY2G20 was inoculated in Landy liquid medium and then cultured for 4 days at 25°C. Salkowski solution was then added to detect IAA. A 50-mg/L IAA solution was used as the positive control. Landy medium without inoculation of strain BY2G20 was the negative control. The red color indicates the existence of IAA. (F) Strain BY2G20 inhibits the growth of R. solanacearum. Single colonies of strain BY2G20 and R. solanacearum were inoculated in LB liquid medium for 12 h at 37°C. R. solanacearum was then mixed with LB solid medium and plated with oxford cups. The culture supernatant of strain BY2G20 was inoculated into the holes of the oxford cups and then cultured for 24 h at 37°C.

The colony morphology of strain BY2G20 on LB medium was round, with a moist surface and regular edges, and appeared creamy yellow (Fig. 1A). The growth temperature range of strain BY2G20 on LB medium was 13 to 46°C, with an optical range of 28 to 42°C (data not shown). Strain BY2G20 could grow at pH 7 to 9, and its salt tolerance concentration was 7% (Fig. 1B and C). The BY2G20 cells were typically rod-shaped and often appeared as connected filaments (Fig. 1D). Through Gram staining, strain BY2G20 became Gram variable (Fig. 1D). According to the results of the carbon source utilization, BY2G20 can use sucrose, maltose, glucose, fructose, and xylose but cannot effectively use lactose, galactose, and l-arabinose. Strain BY2G20 has the ability to secrete IAA, although the secretion amount was not high (Fig. 1E). The antagonistic assay showed that strain BY2G20 could antagonize the phytopathogen Ralstonia solanacearum (Fig. 1F) and exhibited weakly antagonistic ability against *Verticillium dahlia* (see Fig. S1 in the supplemental material).

10.1128/msystems.01426-21.1 FIG S1 The antagonistic ability of strain BY2G20 against V. dahliae. The preserved pathogenic hyphae of V. dahliae were placed in the center of a PDA plate, and single colonies of strain BY2G20 were inoculated around V. dahliae and then cultured for 6 days at 28°C. Download FIG S1, PDF file, 1.9 MB. Copyright © 2022 Yin et al. 2022 Yin et al. https://creativecommons.org/licenses/by/4.0/ This content is distributed under the terms of the Creative Commons Attribution 4.0 International license.

The major fatty acids (>10%) of BY2G20 were iso-C_15:0_ (16.7%), anteiso-C_15:0_ (30.0%), and C_16:0_ (17.7%). A detailed fatty acid profile is shown in Table S1. Large amounts of iso- and anteiso-branched fatty acids are typical of members of the genus *Metabacillus* (11). BY2G20 is similar to *M. lacus* AK74^T^ and *M. indicus* MTCC 4374^T^ in the production of major fatty acids (iso-C_15:0_, anteiso-C_15:0_, and C_16:0_) but differs from *M. mangrovi* AK61^T^ and *M. idriensis* DSM 19097^T^ in the production of compounds (iso-C_14:0_ and iso-C_16:0_).

10.1128/msystems.01426-21.3 TABLE S1 Fatty acid composition of BY2G20 compared to the closely related type strains of the genus *Metabacillus*. Download Table S1, DOCX file, 0.02 MB. Copyright © 2022 Yin et al. 2022 Yin et al. https://creativecommons.org/licenses/by/4.0/ This content is distributed under the terms of the Creative Commons Attribution 4.0 International license.

### BY2G20 belongs to a novel genomospecies of the genus *Metabacillus*.

**FIG 2:**
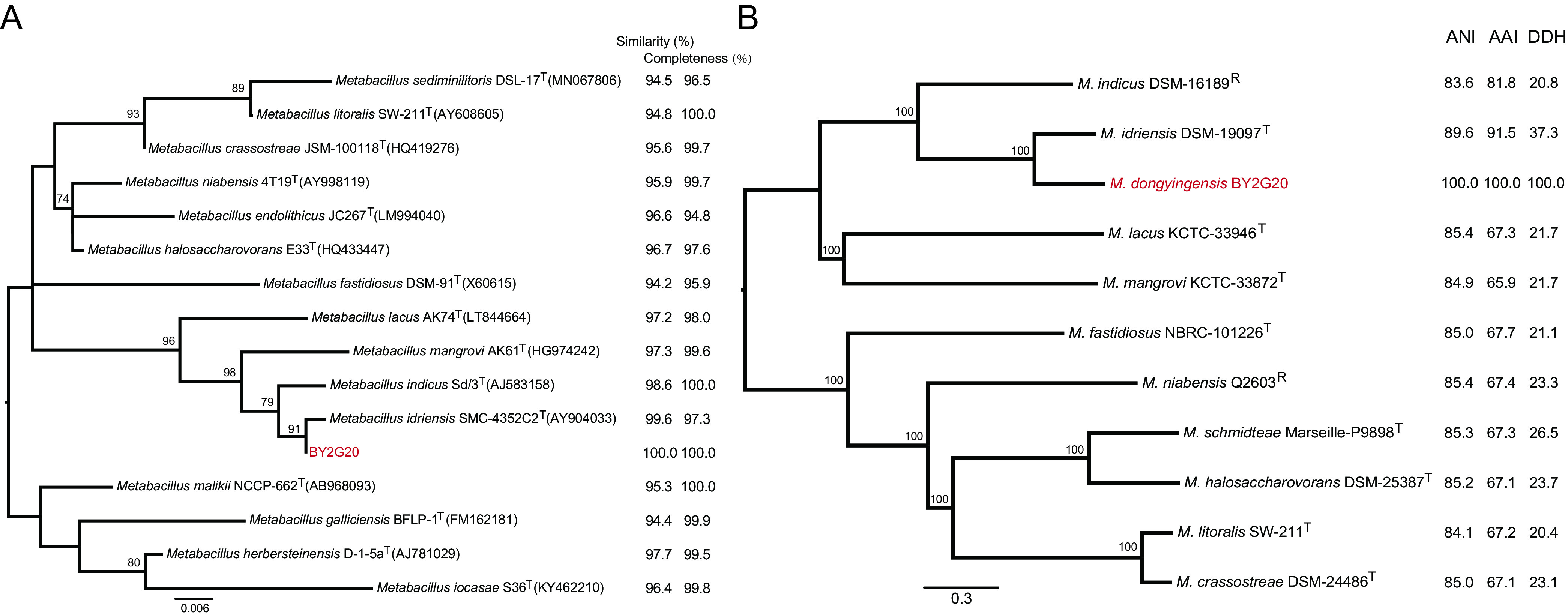
Phylogenetic analysis. (A) Phylogenetic tree based on 16S rRNA sequences obtained by the ML method with 1000 replicates. (B) ML phylogeny was constructed using concatenated SNPs derived from 1,433 single-copy core gene families of 11 *Metabacillus* genomes. The node values of the tree are bootstrap values (1,000 replicates). The values next to the tree indicate ANI, AAI, and *in silico* DDH values. Novel effective species “*M. dongyingensis*” strain BY2G20 was used as a query, and the listed genome was used as a reference. Numbers at nodes indicate the levels of bootstrap support (>70%).

The complete 16S rRNA sequence (1,544 bp) of BY2G20 was subjected to similarity-based searches against the taxonomically united 16S rRNA database in EzBioCloud (12) to identify similar species. All the 16S rRNA sequences (see Table S2) of closely related species were collected from the EzBioCloud and LPSN database and used to construct the phylogenetic tree with the neighbor-joining (NJ), maximum-likelihood (ML), and minimum-evolution methods (Fig. 2A; see also Fig. S2). Strain BY2G20 was closely related to *M. idriensis*, *M. indicus*, *M. mangrovi*, and *M. lacus*, and was in a separate clade within the genus *Metabacillus* based on the phylogenetic trees of 16S rRNA (Fig. 2A; see also Fig. S2). The 16S rRNA sequence showed 99.6, 98.6, 97.3, and 97.2% similarities with the corresponding genes from *M. idriensis* SMC-4352C2^T^, *M. indicus* Sd/3^T^, *M. mangrovi* AK61^T^, and *M. lacus* AK74^T^, respectively.

10.1128/msystems.01426-21.2 FIG S2 Phylogenetic tree based on 16S rRNA sequences obtained by the neighbor-joining (NJ) method (A) and minimum evolution method (B) with 1,000 replicates. Numbers at nodes indicate the levels of bootstrap support (>70%). (C) Comparative analysis of biosynthetic gene clusters for secondary metabolism from BY2G20 and other strains. Different genes are in different colors, and genes with the same color are homologous to each other. Download FIG S2, PDF file, 0.7 MB. Copyright © 2022 Yin et al. 2022 Yin et al. https://creativecommons.org/licenses/by/4.0/ This content is distributed under the terms of the Creative Commons Attribution 4.0 International license.

10.1128/msystems.01426-21.4 TABLE S2 Genetic characteristics of the sequences in the present study. Download Table S2, XLSX file, 0.01 MB. Copyright © 2022 Yin et al. 2022 Yin et al. https://creativecommons.org/licenses/by/4.0/ This content is distributed under the terms of the Creative Commons Attribution 4.0 International license.

To further evaluate its taxonomic position in the genus *Metabacillus*, the complete genome of BY2G20 was sequenced, and 10 public type/reference genomes (see Table S2) of closely related species were collected for subsequent analyses. A high-resolution phylogeny based on 1,433 single-copy core gene families that shared all 11 genomes was generated (Fig. 2B). In the core genome tree, BY2G20, together with *M. idriensis* DSM-19097^T^ and *M. indicus* DSM-16189^R^, formed a monophyletic clade, that was deeply nested within the genus *Metabacillus*. The BY2G20 chromosome shared <90.0% average nucleotide identity (ANI), <92.0% average amino acid identity (AAI), and <38.0% *in silico* DNA-DNA hybridization (DDH) values with other members of *Metabacillus* (Fig. 2B). Based on these results, BY2G20 is most closely related to *M. idriensis* DSM-19097^T^. Despite sharing 99.6% 16S rRNA sequence similarity with *M. idriensis*, the ANI and AAI values shared by BY2G20 and DSM-19097^T^ were 89.6 and 91.5%, respectively, which was much lower than the 94.0% cutoff value proposed previously for different *Bacillus* species (13). The *in silico* DDH value shared by BY2G20 and DSM-19097^T^ was 37.3% (34.8 to 39.8%; 1.3% probability DDH ≥ 70%), which was also much lower than the 70% species threshold (14). Thus, 16S rRNA and core genome phylogenetic analysis combined with genomic properties indicated that BY2G20 should be considered a novel genomospecies in the genus *Metabacillus*, for which the name *Metabacillus dongyingensis* sp. nov. is proposed. The type strain is BY2G20^T^ (CGMCC 22867^T^).

### Genomic characteristics of BY2G20.

**FIG 3:**
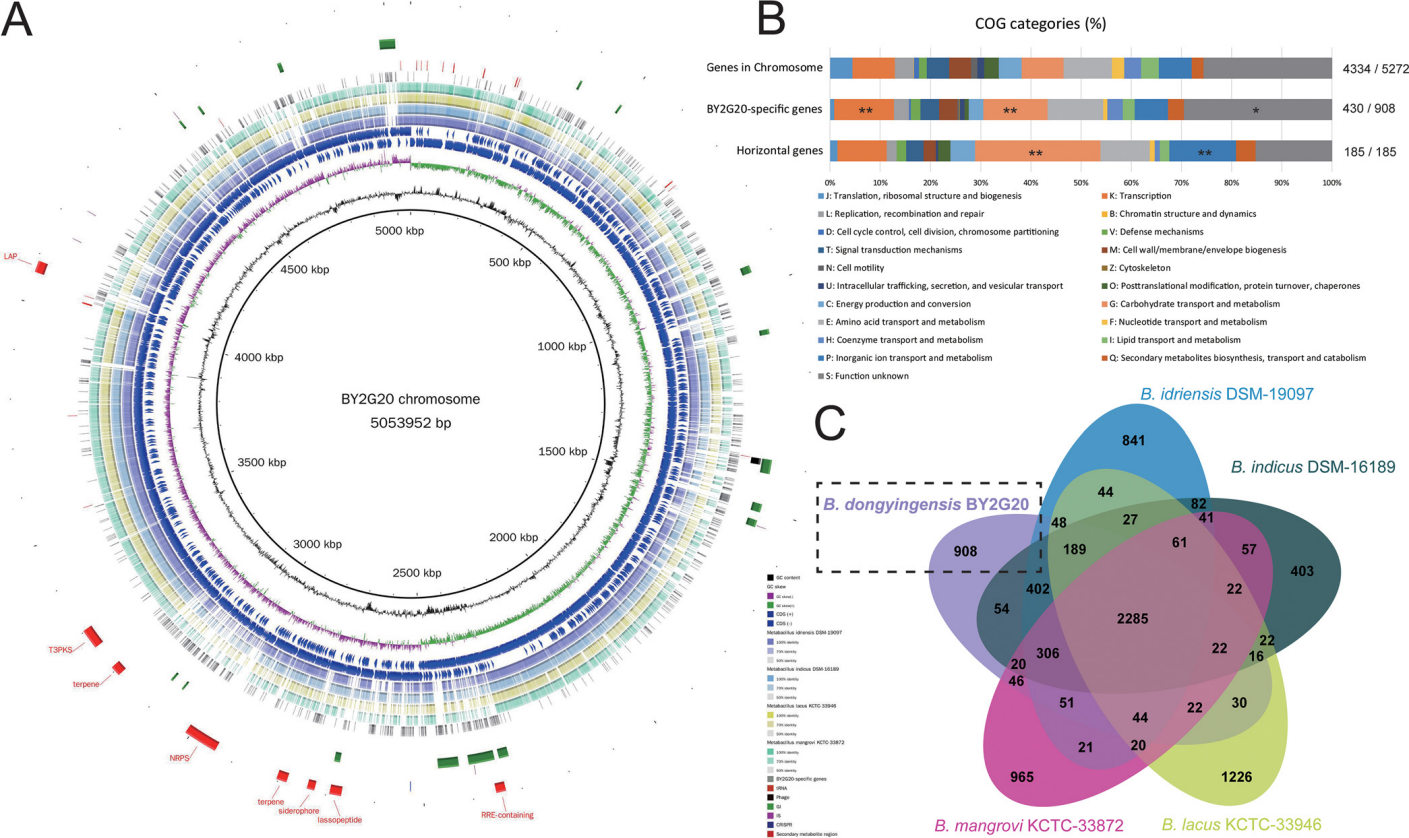
Genome features of the *M. dongyingensis* BY2G20 chromosome. (A) Circular representation of the BY2G20 chromosome. Rings represent the following features labeled from inside to outside: ring 1, GC content; ring 2, GC-skew, green and purple correspond to above- and below-average GC skew, respectively; rings 3 and 4, blue arrows correspond to plus-strand CDS and minus-stand CDS; rings 5 to 8, circular comparison of *M. idriensis* DSM-19097^T^, *M. indicus* DSM-16189^T^, *M. mangrovi* KCTC-33872^T^, and *M. lacus* KCTC-33946^T^, respectively; rings 9 to 13, blocks correspond to BY2G20-specific genes, tRNA, prophage, GIs, ISs, CRISPRs, and secondary metabolite regions, respectively. (B) Distribution of COG categories for chromosome genes, BY2G20-specific genes, and horizontal genes, respectively. *, Fisher exact test *P* value < 0.05; **, Fisher exact test *P* value < 0.01. (C) Venn diagram displaying overlaps and differences in orthologous gene families in BY2G20 and closely related strains.

The complete genome of BY2G20 is comprised of one chromosome and one plasmid, and was deposited in the NCBI GenBank database (accession number GCA_019933155.1). The chromosome of BY2G20 is a circular molecule of 5,053,952 bp, with an average GC content of 40.1% and 5272 RAST-predicted coding sequences (CDSs). The general chromosomal properties of BY2G20 are shown in Fig. 3A. To provide a deeper understanding of the functional distribution of BY2G20, we annotated the CDSs in the chromosome in detail using the Clusters of Orthologous Groups (COGs) assignment. A total of 4334 (82.2%) CDSs were classified into COG families composed of 21 categories (Fig. 3B). The results revealed that “K: transcription” (401 genes), “G: Carbohydrate transport and metabolism” (390 genes), and “E: Amino acid transport and metabolism” (455 genes) were the most enriched functional categories in the chromosome. A high proportion of CDSs (40.6%) was poorly characterized (“S: Functional unknown”: 1,203 genes; “no homologs identified”: 938 genes).

### Genetic plasticity and genomic evolution of BY2G20.

Mobile genetic elements (MGEs) can mediate DNA acquisition and facilitate the transmission of genetic material between different bacterial taxa (15). Several types of MGEs were identified in the BY2G20 genome, including insert sequences (ISs), prophages, genomic islands (GIs), and plasmids (see Table S3). A total of 17 GIs were identified, covering 301.9 kb (5.6%) of the genomic region. The BY2G20 chromosome harbored one incomplete prophage, which was similar to PHAGE_Bacill_Mgbh1 (NC_041879), with a size of 14 kb and GC content of 34.7%. Notably, the prophage and some GIs are absent in other closely related species (Fig. 3A). A total of nine complete IS elements were identified, including five on the chromosome and four on the plasmid. Furthermore, the BY2G20 genome also showed the presence of 84 tRNA codons. These results demonstrate the genetic plasticity of BY2G20.

10.1128/msystems.01426-21.5 TABLE S3 Features of prophages, CRISPRs, tRNA, genomic islands, insertion sequences, and secondary metabolite regions in BY2G20. Download Table S3, XLSX file, 0.02 MB. Copyright © 2022 Yin et al. 2022 Yin et al. https://creativecommons.org/licenses/by/4.0/ This content is distributed under the terms of the Creative Commons Attribution 4.0 International license.

CRISPR-Cas is a general defense system that protects bacteria from foreign DNA (16). In the BY2G20 genome, one CRISPR locus with 25 different spacer sequences was identified (see Table S3). However, this CRISPR locus was not associated with *cas* genes. The presence of the CRISPR-Cas system indicates an advantage in promoting the genome stability of BY2G20 by acting as a barrier to the entry of soil phages.

Horizontal gene transfer (HGT) is the driver for bacterial genetic innovation and speciation and promotes the acquisition of new physiological functions, which is crucial for rapid adaptation to changing niches (15). We identified 185 potential horizontal genes (see Table S4) in the BY2G20 chromosome, many of which are associated with “G: Carbohydrate transport and metabolism” (Fisher exact test *P* value < 0.01) and “P: Inorganic ion transport and metabolism” (Fisher exact test *P* value < 0.01). It can be assumed that the acquisition of the molecular toolbox, comprising genetic elements derived from other soil- and plant-associated bacteria unrelated to *Bacillaceae*, has enhanced the ability of BY2G20 to exploit plant-derived polysaccharides and adapt to saline-alkaline soil.

10.1128/msystems.01426-21.6 TABLE S4 List of potential horizontal genes in the BY2G20 chromosome. Download Table S4, XLSX file, 0.03 MB. Copyright © 2022 Yin et al. 2022 Yin et al. https://creativecommons.org/licenses/by/4.0/ This content is distributed under the terms of the Creative Commons Attribution 4.0 International license.

### Comparative genomic analysis of BY2G20 with closely related species revealed its evolution and speciation.

The exploration of the evolutionary dynamics of the BY2G20 genome will provide insights into the speciation of the species “*M. dongyingensis*.” Thus, we performed a comparative genome analysis of BY2G20 with closely related species. Four strains—*M. idriensis* DSM-19097^T^, *M. indicus* DSM-16189^R^, *M. mangrovi* KCTC-33872^T^, and *M. lacus* KCTC-33946^T^—were used as reference genomes. The BY2G20-specific gene repertoire was characterized, which consisted of gene families that were present in the BY2G20 chromosome and absent in the closely related species. A total of 908 BY2G20-specific gene families (915 genes) were identified (Fig. 3C; see also Table S5). The COG classification showed that the genes unique to BY2G20 were enriched in “K: Transcription,” “G: Carbohydrate transport and metabolism” (Fisher exact test *P* value < 0.01), and “S: Function unknown” (Fisher exact test *P* value = 0.0285). These results suggest a preference toward the metabolism and transport of carbohydrates. As shown in Fig. 3A, additional features in some genes, such as adjacent tRNAs and ISs, as well as remnants of GIs and prophages, indicated the occurrence of HGTs. It can be assumed that HGTs were important evolutionary forces that contributed to the evolution and speciation of *M. dongyingensis* and facilitated bacterium-plant interactions.

10.1128/msystems.01426-21.7 TABLE S5 List of BY2G20-specific genes. Download Table S5, XLSX file, 0.06 MB. Copyright © 2022 Yin et al. 2022 Yin et al. https://creativecommons.org/licenses/by/4.0/ This content is distributed under the terms of the Creative Commons Attribution 4.0 International license.

**FIG 4:**
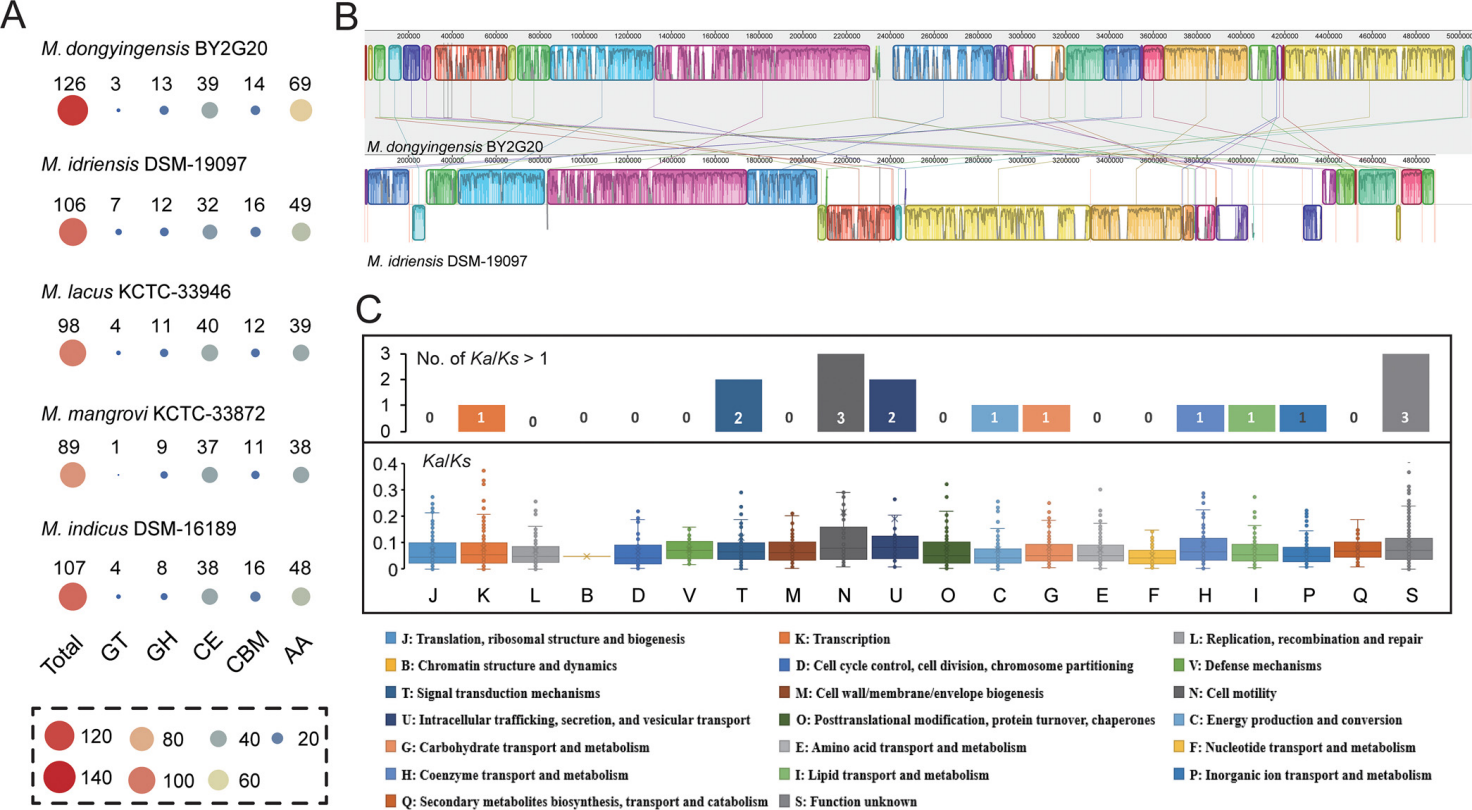
Comparative genomic analysis of BY2G20 and closely related strains. (A) Distribution of CAZymes in BY2G20 and closely related strains. (B) Genome alignment of BY2G20 and *M. idriensis* DSM-19097^T^. Synteny blocks are shown as identically colored regions and are linked across the sequences. Regions inverted relative to the BY2G20 chromosome are shifted downward from the axis. (C) Distribution of the *Ka*/*K_s_* rates of the pairwise orthologs from BY2G20 and *M. idriensis* DSM-19097^T^ in COG functional categories.

CAZymes were further identified to understand the mechanism of polysaccharide hydrolysis in BY2G20. The BY2G20 genome contained an abundance of CAZyme-encoding genes for carbohydrate esterases (CEs; *n* = 39) and auxiliary activities (AAs; *n* = 69), and a small number of genes encoding glycoside hydrolases (GHs; *n* = 3), glycosyltransferases (GTs; *n* = 13), and carbohydrate binding molecules (CBMs; *n* = 14). Compared to its closely related species, BY2G20 harbored more CAZyme-encoding genes (*n* = 126). The expanded gene families were mainly involved in AAs (Fig. 4A). Furthermore, BY2G20 had 16 strain-specific CAZyme-encoding genes (GHs, 13; GTs, 2; and CBMs, 1). These abundant CAZyme-encoding genes might contribute to the metabolic diversity of BY2G20 in diverse environments, particularly in the rhizosphere.

### Evolutionary dynamics between BY2G20 and *M. idriensis* DSM-19097^T^.

To further elucidate the evolutionary dynamics of BY2G20, the genomes of BY2G20 and *M. idriensis* DSM-19097^T^ were compared. Genome rearrangement and synteny indicate evolutionary relationships between genomes. Synteny blocks also called locally colinear blocks, represent genomic regions comprising the genes that are orthologous and co-arranged with the compared genome (17). Synteny analysis revealed numerous synteny blocks in BY2G20 and DSM-19097^T^, spanning 2,976,363 bp (58.9%) and 2,975,715 bp (60.9%), respectively (Fig. 4B). About 40% of the genome regions were not in the syntenic blocks. As shown in Fig. 4B, the synteny blocks were considerably fragmented, and most of these blocks were not long. Our results suggest that dramatic rearrangements occurred in the genomes of BY2G20 and *M. idriensis* DSM-19097^T^ after they diverged from their most recent common ancestor.

To pinpoint the critical genetic function changes between BY2G20 and DSM-19097^T^, the evolutionary signatures of 2,835 orthologous gene pairs were characterized by their nonsynonymous (*K_a_*) and synonymous (*K_s_*) substitution rates (*K_a_*/*K_s_*). The *K_a_*/*K_s_* rates of most of the pairwise orthologs (*n* = 2,814; 99.3%; average *K_a_*/*K_s_ *=* *0.084 ± 0.07) were <1, indicating a predominant action of purifying selection. The purifying selection suggested functional constraints that would maintain a stable and adapted genomic core. We further investigated the degree of constraint of each functional category. Genes involved in the “F: Nucleotide transport and metabolism” (average *K_a_*/*K_s_ *=* *0.052 ± 0.001), “P: Inorganic ion transport and metabolism” (average *K_a_*/*K_s_ *=* *0.072 ± 0.001), “L: Replication, recombination and repair” (average *K_a_*/*K_s_ *=* *0.071 ± 0.006), and “D: Cell cycle control, cell division, chromosome partitioning” (average *K_a_*/*K_s_ *=* *0.069 ± 0.004) exhibited stronger evolutionary constraints (Fig. 3C). In contrast, genes related to “N: Cell motility” (average *K_a_*/*K_s_ *=* *0.216 ± 0.240) and “U: Intracellular trafficking, secretion, and vesicular transport” (average *K_a_*/*K_s_ *=* *0.193 ± 0.222) underwent weaker evolutionary constraints (Fig. 4C). The different degrees of purifying selection operating on functional genes exhibited by the difference in the *K_a_*/*K_s_* rates suggested that the evolutionary strategies may be carried out under different constraints. A total of 21 pairwise orthologs were identified as positively selected (*K_a_*/*K_s_*> 1) (see Table S6). Similarly, the positively selected genes were involved in several functional categories, including “T: Signal transduction mechanisms” (*n* = 2), “N: Cell motility” (*n* = 3), and “U: Intracellular trafficking, secretion, and vesicular transport” (*n* = 2) (Fig. 4C). Considering the different niches of two strains (BY2G20 isolated from saline-alkaline soils; the selected DSM-19097^T^ isolated from Homo sapiens), it can be inferred that the genes involved in “inorganic ion transport and metabolism” keep their functions in saline-alkaline environments, and the weaker functional constraints of “cell motility” promote adaptation during habitat conversion.

10.1128/msystems.01426-21.8 TABLE S6 List of positively selected pairwise orthologs (*K_a_*/*K_s_ *>* *1) between BY2G20 chromosome and *M. idriensis* DSM-19097^T^. Download Table S6, XLSX file, 0.01 MB. Copyright © 2022 Yin et al. 2022 Yin et al. https://creativecommons.org/licenses/by/4.0/ This content is distributed under the terms of the Creative Commons Attribution 4.0 International license.

### Plasmid analyses revealed that the genetic exchanges between plasmid and chromosome might promote adaptation to the rhizosphere.

**FIG 5:**
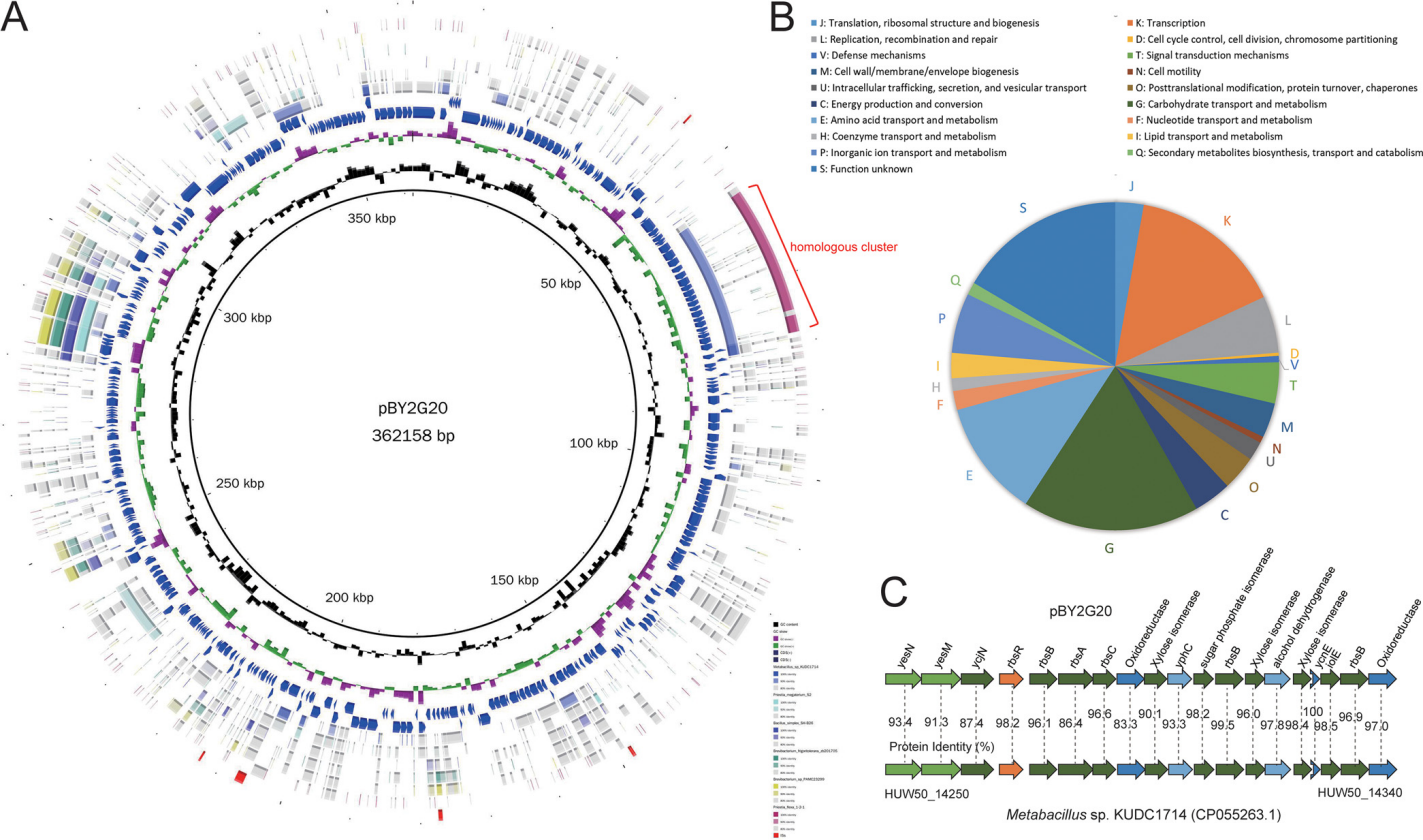
Structure and recombination analysis of the plasmid pBY2G20. (A) Circular comparison between the plasmid pBY2G20 and other reported similar genomes. Rings represent the following features labeled from inside to outside: ring 1, GC content; ring 2, GC-skew; rings 3 and 4, blue arrows correspond to plus-strand CDS and minus-stand CDS; rings 5 to 8, circular comparison of *Metabacillus* sp. KUDC1714, *Priestia megaterium* S2, Bacillus simplex SH-B26, Brevibacterium frigoritolerans zb201705, *Brevibacterium* sp. PAMC23299, and *Priestia flexa* 1-2-1, respectively; ring 9, blocks correspond to ISs. (B) Distribution of COG categories for plasmid genes. (C) Genetic organization and comparison of the homologous region between pBY2G20 and the chromosome of *Metabacillus* sp. KUDC1714. Color coding for genes is based on COG categories. The percent amino acid identities of each homolog are shown.

This strain BY2G20 harbors a plasmid, pBY2G20, comprising 362,158 bp with 384 CDSs (Fig. 5A). The plasmid makes up 6.7% of the BY2G20 genome and has a lower GC content than that of the chromosome (36.7% versus 40.1%). The plasmid also carries a variety of functional genes, including genes related to “K: transcription” (49 genes), “G: Carbohydrate transport and metabolism” (56 genes), “E: Amino acid transport and metabolism” (37 genes), and “P: Inorganic ion transport and metabolism” (19 genes) (Fig. 5B). These transport and metabolic genes on the plasmid are organized in what appears to be functional operon structures, which may enable this strain to survive in rhizosphere habitats. The poorly characterized CDSs (“S: Functional unknown”, 53 genes; “no homologs identified”, 94 genes) make up 38.3% of the plasmid.

A BLASTn search of the NCBI Non-Redundant (NR) database using the full plasmid gene sequences was performed. Interestingly, the five sequences with the largest number of homologs were the chromosomes of strains associated with soil habitat (see Table S1), including *Metabacillus* sp. KUDC1714 (57 homologs), *Priestia megaterium* S2 (47 homologs), *Brevibacterium* sp. PAMC23299 (37 homologs), Brevibacterium frigoritolerans ZB201705 (35 homologs), and Bacillus simplex SH-B26 (35 homologs). Despite the small number of homologous genes, some genes shared more than 90% identity between pBY2G20 and their chromosomal homologs. These homologous genes were scattered in different locations of pBY2G20. Nineteen homologous genes clustered together and appeared to have been cotransferred (Fig. 5A and C). They exhibited consistent organizations of gene loci and presented approximately 95% identity of protein sequences between the plasmid and chromosome (Fig. 3C). A similar homologous region was also found in the chromosome of *Priestia flexa* 1-2-1. This set of genes mainly encodes carbohydrate transport and metabolism-related functions, such as the two-component system YesM-YesN, the ABC transporter complex RbsABC for ribose import (18), sugar isomerase, and oxidoreductase. These results revealed the genetic exchange between the plasmid and chromosome, and highlighted the role of pBY2G20 in metabolic activities and strain-specific niche adaptations.

### Genomic adaptations of BY2G20 to saline-alkaline soil.

**FIG 6:**
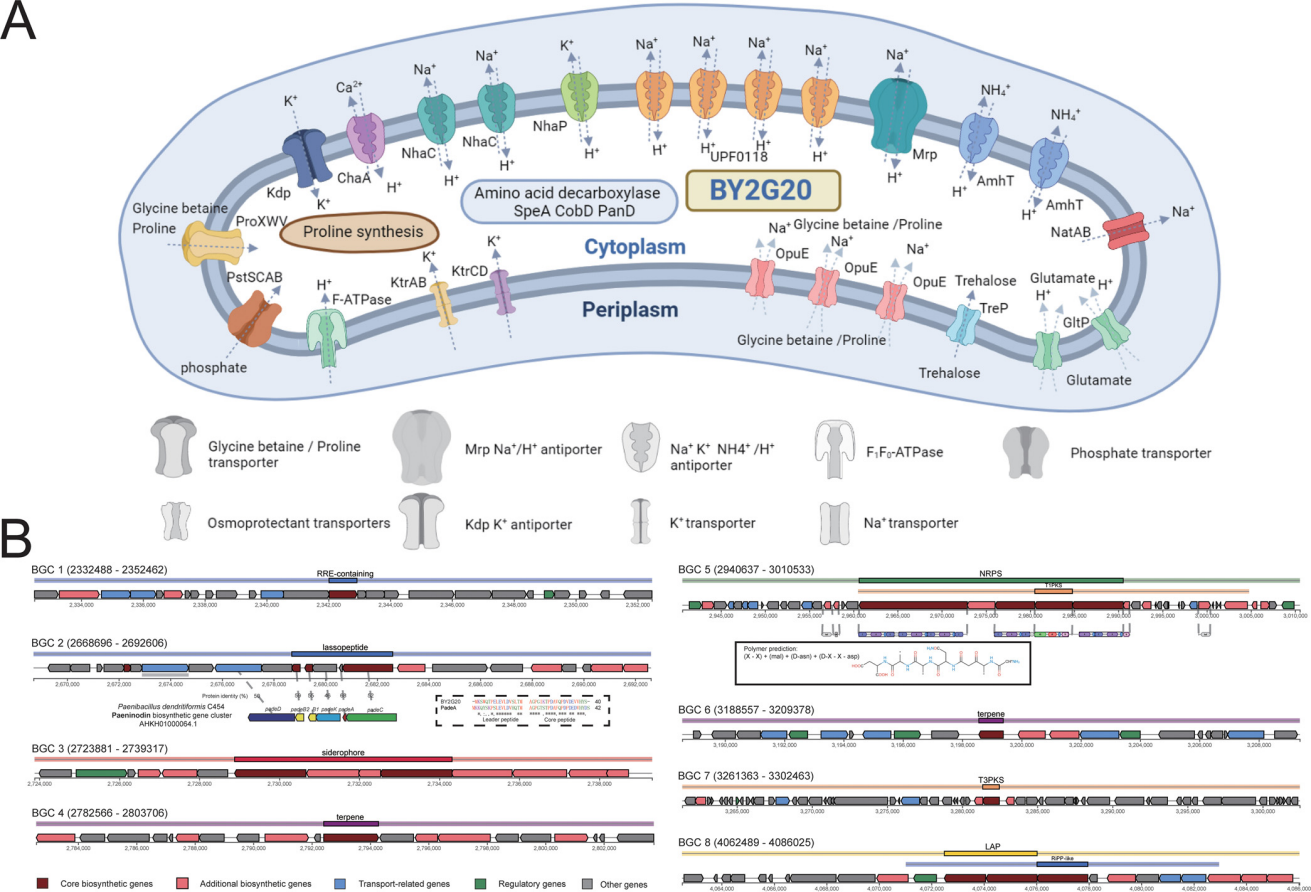
Genomic characteristics of BY2G20 to salt and alkali tolerance and secondary metabolism. (A) Cell cartoon constructed from the genome annotation of BY2G20. Features putatively involved in the adaptation to high alkalinity and salinity are shown. (B) Genetic organization of the gene clusters for secondary metabolism in the BY2G20 chromosome. The region in the inset dotted box shows the comparison of the amino acid sequences of the leader peptide and core peptide of secondary metabolite region 2 and paeninodin biosynthetic gene cluster. The region in the inset box shows the predicted monomer structures for secondary metabolite region 5 (NRPS).

BY2G20 was isolated from saline-alkaline soil. The experimental results showed that it was adapted to saline (0 to 7% NaCl) and alkaline (pH 7 to 9) conditions (Fig. 1B and C). At high pH, alkaliphilic and alkalitolerant microbes are able to maintain a near-neutral cytoplasmic pH to meet pH challenges (5, 19). The following comparative genomic analysis of BY2G20 identified several features that are likely to be involved in its salt and alkali tolerance (Fig. 6A).

### Cation/proton antiporters.

The activation of key cation/proton antiporters for inward proton transport is an important adaptation mechanism for pH homeostasis under alkaline conditions (20–22). The BY2G20 genome harbored a set of genes coding for cation/proton antiporters, including Na^+^/H^+^ antiporters, K^+^/H^+^ antiporter, Ca^2+^/H^+^ antiporter, and NH_4_^+^/H^+^ antiporters (see Table S7). Na^+^/H^+^ antiporters comprised Mrp-type antiporters encoded by the *mrpA-G* operon, two copies of NhaC-type antiporters, and four copies of UPF0118-family antiporters. Mrp, NhaC, and NhaH antiporters have been reported to be crucial for growth under saline and/or alkaline conditions in alkaliphilic microbes because they maintain a lower cytosolic pH compared to the environment by exporting Na^+^ and importing H^+^ (20, 23). UPF0118 and its homologs were found to exhibit pH-dependent Na^+^(Li^+^)/H^+^ antiport activity in Halobacillus andaensis NEAU-ST10-40^T^; a moderate halophile that can tolerate up to 15% NaCl (24, 25). Furthermore, one *nhaP*, one *chaA*, and two copies of *amhT* present in the BY2G20 genome encode a K^+^/H^+^ antiporter, a Ca^2+^/H^+^ antiporter, and two NH_4_^+^/H^+^ antiporters, respectively. These antiporters allow the bacteria to avoid excessive cation accumulation by importing H+ while simultaneously pumping out K^+^, Ca^2+^, and NH_4_^+^. In particular, ammonium homeostasis is crucial at high pH because cytosolic ammonium accumulation interferes with cytoplasmic pH regulation (26).

10.1128/msystems.01426-21.9 TABLE S7 List of genes identified in the BY2G20 genome with predicted functions in adaptation to high alkalinity and salinity and Trp-dependent IAA production. Download Table S7, XLSX file, 0.02 MB. Copyright © 2022 Yin et al. 2022 Yin et al. https://creativecommons.org/licenses/by/4.0/ This content is distributed under the terms of the Creative Commons Attribution 4.0 International license.

### Na^+^ transporter.

The Na^+^ transporter also has a major physiological role in Na^+^ resistance and Na^+^-dependent pH homeostasis. The *natAB* operon present in BY2G20 produces an ABC transport system that catalyzes ATP-dependent electrogenic Na^+^ extrusion without mechanistically coupled K^+^ or H^+^ uptake. Na^+^ is more toxic at high pH for growth than at neutral pH. The NatAB transporter is crucial for Na^+^ resistance at high pH in B. subtilis JC901 (27). Furthermore, the *natAB* operon was absent in the *M. idriensis* DSM-19097^T^ genome. Thus, the NatAB transporter is presumably a crucial component of the Na^+^ resistance capacity of BY2G20 at high pH.

### K^+^ uptake.

Many alkaliphilic microbes maintain an inward-directed K^+^ gradient since cytoplasmic K^+^ accumulation may compensate for Na^+^ toxicity at high pH (28, 29). Furthermore, the stimulation of K^+^ uptake is the most rapid response to an osmotic upshock in microbes (30). The BY2G20 genome harbored a set of genes encoding one KtrAB transporter, one KtrCD transporter, and one Kdp-type transporter. The K^+^ uptake systems of KtrAB and KtrCD in B. subtilis have a major physiological role in adaptation to hypertonicity (31). Kdp transporter, as a high-affinity K^+^ uptake system, is important at low potassium concentrations (30). Noticeably, the *kdp* operon was absent in the *M. idriensis* DSM-19097^T^ genome.

### Osmoprotectant transport and synthesis.

Glycine betaine, proline, glutamate, and trehalose are effective osmoprotectants, and their cytoplasmic accumulation protects microbes under high-salt and osmotic stress (32, 33). The *proVWX* operon encoding an ABC transporter for a high-affinity glycine betaine and proline uptake was found in the BY2G20 genome (34). Furthermore, the *opuA* and *opuBC* genes were identified (see Table S7). They also encode an ABC transporter for glycine betaine uptake (35). Three copies of *opuE* genes present in BY2G20, encoding a Na^+^ symporter, obligatorily couple proline uptake to Na^+^ symport (33). OpuE-mediated proline uptake is crucial for growth in a high-osmolarity environment in B. subtilis (33). Furthermore, two copies of the *proAB* operon with one *proC* gene were found in the BY2G20 genome and might contribute to proline biosynthesis (36). For glutamate uptake, two copies of the *gltP* gene encoding the H^+^-glutamate symport protein were identified (37). For trehalose uptake, one trehalose phosphotransferase system encoded by the *treP* gene was present in BY2G20 (38). Thus, osmoprotectant uptake and synthesis might serve as an important feature of BY2G20 in adaptation to saline-alkaline soil.

### Other genomic characteristics to saline-alkaline adaptation.

Cytoplasmic pH has also been reported to be maintained by the metabolism of proton buffer molecules and the expression of amino acid decarboxylase (5). We found a *pstSCAB* operon for phosphate uptake, *speA* encoding arginine decarboxylase, *cobD* coding threonine decarboxylase, and *panD* encoding aspartate decarboxylase in the BY2G20 genome (see Table S7). Furthermore, the *atpA-G* operon was identified. These genes encode H^+^-transporting F_1_F_0_-ATPase, which imports H^+^ during ATP synthase activity and will be induced under alkaline conditions (23).

Comparisons of the genomic characteristics to saline-alkaline adaptation between BY2G20 and *M. idriensis* DSM-19097^T^ yielded significantly lower average *K_a_*/*K_s_* values (0.052 ± 0.034; *t* test *P* value < 0.01) compared to that of the core genome (average *K_a_*/*K_s_ *=* *0.084 ± 0.07), indicating the predominant action of purifying selection. The genes with lower *K_a_*/*K_s_* values have a stronger tendency to keep their functions (39). It can be inferred that the genes adapted to saline-alkaline ecosystems under functional constraints maintained a stable and adapted genomic characteristic for BY2G20.

### Plant growth-promoting characteristics and genetic basis of BY2G20.

PGPRs can be used as soil inoculants in agriculture and horticulture for plant growth promotion and biocontrol. As described below, we performed *in vitro* experiments and genomic analysis to screen plant growth-promoting traits in BY2G20 under salt stress.

### IAA production.

Strain BY2G20 has a weak ability to secrete IAA (Fig. 1E). Bacterial IAA synthesis is mainly from Trp, which can occur via four main alternative pathways: the indole-3-pyruvic acid (IPyA), indole-3-acetamide (IAM), indole-3-acetonitrile (IAN), and tryptamine (TAM) pathways (40, 41). The BY2G20 genome was used to mine genes likely involved in IAA biosynthesis. In BY2G20, we identified the putative gene *aldH* (ORF208 and ORF2922) encoding indole-3-acetaldehyde (IAAld) dehydrogenase of the final step in both the IPyA and TAM pathways. For the TAM pathway, only the putative gene *mao* (ORF2388) encoding monoamine oxidase was identified (see Table S7). The nitrilase gene *yhcX* (ORF771), which is involved in the IAN pathway of *B. velezensis* SQR9 IAA biosynthesis (41), was also found in the BY2G20 genome. Nevertheless, no complete pathways were detected, indicating the limited synthetic capability of IAA synthesis in BY2G20.

### Antagonizing phytopathogen and secondary metabolite production.

The antagonistic assay (Fig. 1F) showed that strain BY2G20 could antagonize the plant bacterial wilt pathogen R. solanacearum. Secondary metabolites are versatile weapons that bacteria use to fight other microbes and protect host plants from microbial pathogens (7). Genome mining analysis identified eight putative biosynthesis gene clusters (BGCs) responsible for the production of potential secondary metabolites (Fig. 3A), including RRE-containing substrate (BGC-1), lassopeptide (BGC-2), siderophore (BGC-3), terpene (BGC-4 and -6), NRPS-T1PKS hybrid (BGC-5), T3PKS (BGC-7), and LAP-RiPP-like hybrid (BGC-8) (Fig. 6B). Six gene clusters (BGC-2, -3, -4, -5, -7, and -8) were identified that contain the homologous genes from known clusters in other *Bacillus* strains (see Fig. S2C). It is most noticeable that 35% of genes in BGC-5 showed similarities to paenilamicin, which is reported to have antibacterial, antifungal, and cytotoxic activities (42). Most genes in BGC-5 exhibited BY2G20 specificity and no homologs in other closely related species (Fig. 3A). This novel NRPS-PKS hybrid cluster plays a possible role in BY2G20 in plant disease biocontrol. Furthermore, BGC-1 and -6 did not show similarities to those present in the antiSMASH database. These diversities of BGCs could be driven by the evolution of BY2G20 to survive and adapt under rhizosphere conditions.

### The growth-promoting capacity of strain BY2G20 for maize under salt stress on nutrient solution.

**FIG 7:**
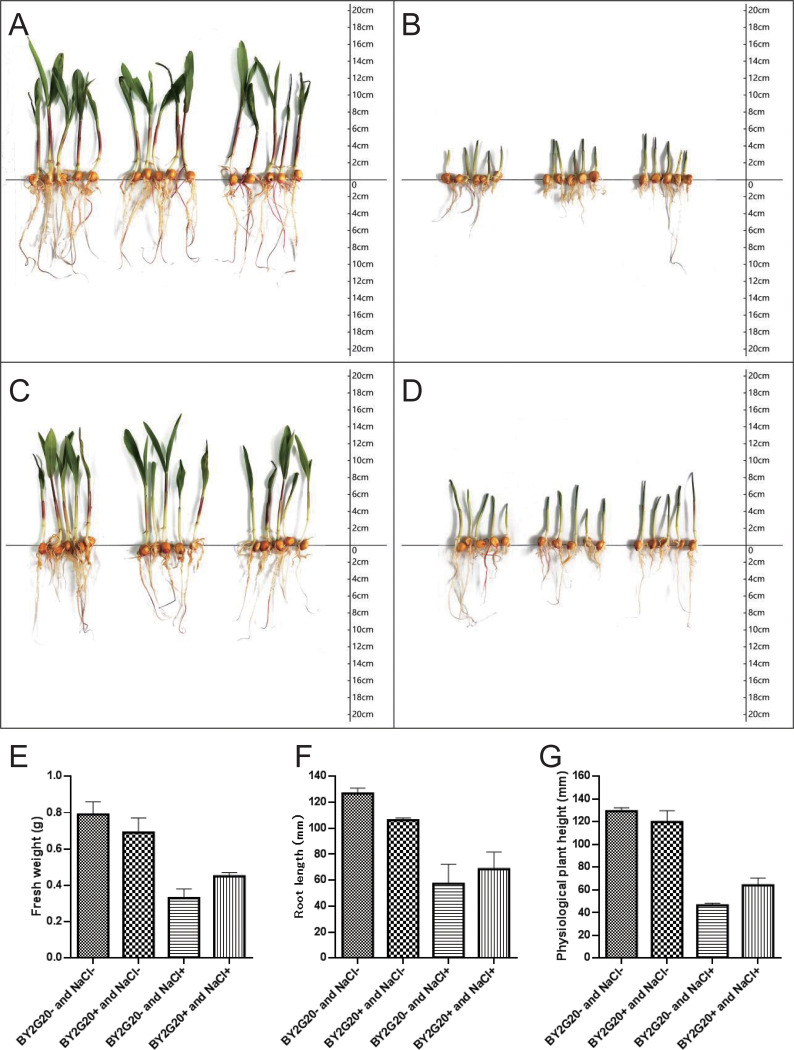
Effects of strain BY2G20 on maize growth on nutrient solution. The maize seedlings were grown for 8 days on half-strength Hoagland’s nutrient solution supplemented with 100 mM NaCl or not, after being dipped in liquid containing strain BY2G20 at a concentration of 10^6^ CFU/mL. Diagram of seedling growth status: (A) BY2G20^−^ and NaCl^−^, (B) BY2G20^−^ and NaCl^+^, (C) BY2G20^+^ and NaCl^−^, (D) BY2G20^+^ and NaCl^+^. (E to G) Physiological plant heights (E), root lengths (F), and fresh weights (G) of maize seedlings.

On day 4 of culture, strain BY2G20 showed an early inhibitory effect on maize growth (data not shown). On day 8 of culture (Fig. 7), the addition of strain BY2G20 still inhibited the growth of maize under salt-free conditions, and the physiological plant height, root length, and fresh weight of maize were reduced by 7.4% (*t* test *P < *0.01), 16.3 (*t* test *P < *0.01), and 12.8%, respectively. However, in the presence of 100 mM NaCl, the physiological plant height, root length, and fresh weight of the seedlings were increased by 37.9% (*t* test *P < *0.05), 19.7%, and 37.1% (*t* test *P < *0.01), respectively. On day 12 of growth, strain BY2G20 still showed the same growth-promoting trend under high-salt conditions. Meanwhile, under salt-free conditions, strain BY2G20 could also increase the maize root length and fresh weight by 16.4 and 5.1%, respectively, although not significantly (data not shown). Thus, strain BY2G20 could overall promote maize growth under saline conditions based on the experiment on nutrient solution. This growth-promoting capacity of strain BY2G20 might be related to its saline-alkaline soil habitat and the identification of genes for salt tolerance.

### Growth-promoting capacity of strain BY2G20 for maize in the soil.

**FIG 8:**
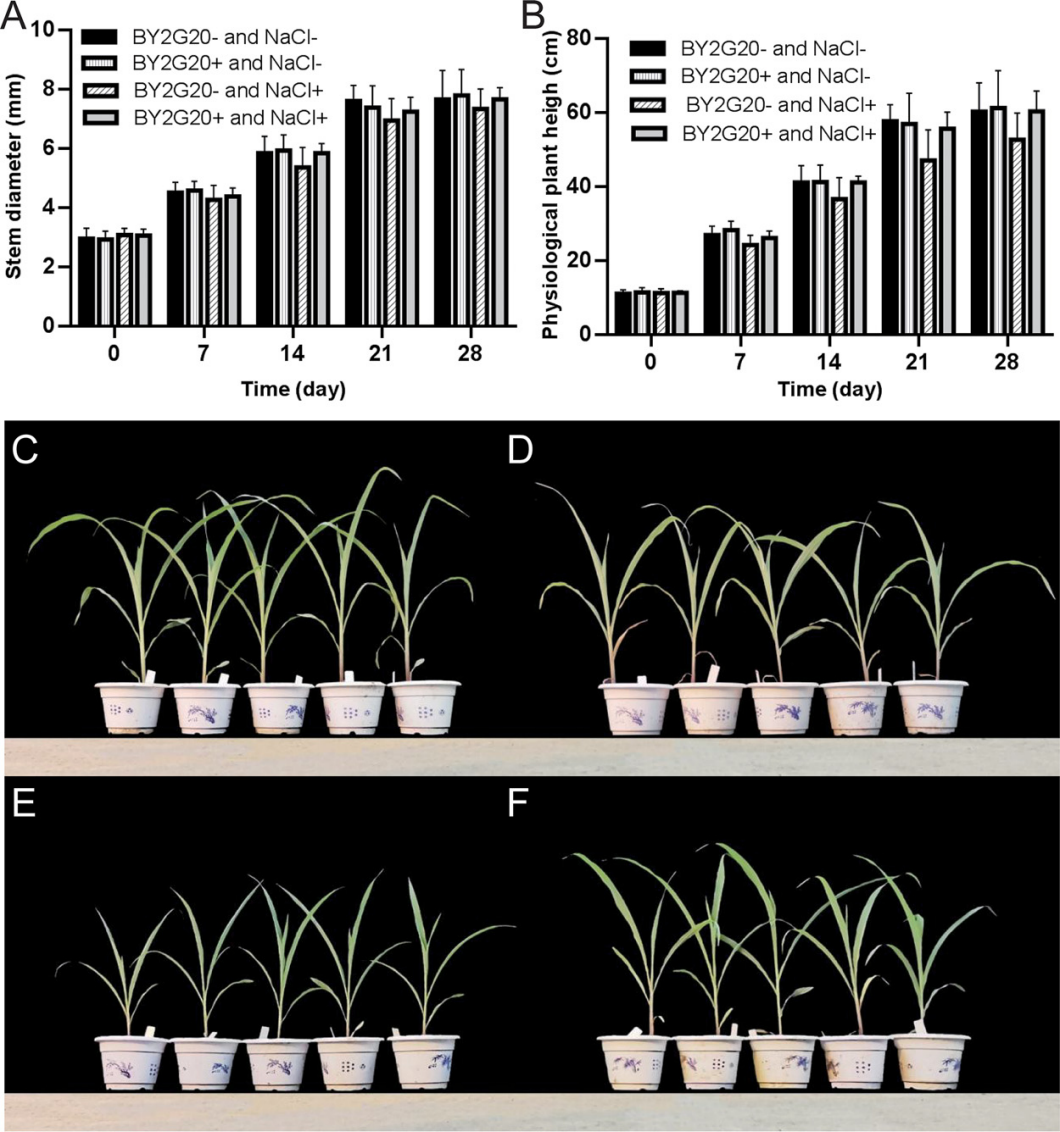
The growth-promoting capacity of strain BY2G20 for maize in the soil environment. The stem diameter (A) and physiological plant height (B) of maize seedlings at different growth days. States of maize growth at 28 days under different treatments: (C) BY2G20^−^ and NaCl^−^, (D) BY2G20^+^ and NaCl^−^, (E) BY2G20^−^ and NaCl^+^, (F) BY2G20^+^ and NaCl^+^. Uniformly sized maize seeds were selected, sterilized for planting, and then thinned at the two-leaf stage. A 200-mL volume of 100 mM NaCl was irrigated 2 days after thinning in the NaCl-treated groups, and the other two groups were irrigated with an equal amount of sterile water. Two days after NaCl treatment, a volume of 200-mL solution containing 10^6^ CFU/mL strain BY2G20 was applied to the maize plants in the strain treated groups, and the other two groups were irrigated with an equal amount of sterile water.

The growth-promoting capacity of strain BY2G20 on maize under salt stress was further studied in a pot experiment. After applying strain BY2G20, the stem diameter and physiological plant height of the maize seedlings were measured every 7 days. On day 28, the aboveground/belowground dry and fresh weights were measured (Fig. 8).

Supplementation of BY2G20 had a promoting effect on the maize stem diameter and physiological plant height under high-salt conditions; however, no effect was observed under the salt-free condition (Fig. 8A and B). Under high salt conditions, treatment with BY2G20 exhibited a slightly promoting effect on the stem diameter on days 7, 21, and 28, while an 8.9% increase (*t* test, *P < *0.05) was detected on day 14 compared to the aseptic group. In terms of physiological plant height, the group treated with strain BY2G20 had an 8.1% increase (*t* test, *P < *0.05) on day 7. This improvement was enhanced on days 14, 21, and 28, showing 12.3, 18.1, and 14.6% increases, respectively (*t* test, *P < *0.01).

The seedlings were harvested on day 28 of culture. Under salt-free conditions, strain BY2G20 did not play a role in promoting maize growth (Fig. 8C and D). Under high-salt conditions, the aboveground and belowground fresh weights of maize treated with BY2G20 increased by 45.4% (*t* test, *P < *0.01) and 4.2%, respectively. Furthermore, treatment with BY2G20 increased the aboveground and belowground dry weights of maize by 28.0% (*t* test, *P < *0.05) and 8.5%, respectively. The presence of strain BY2G20 could significantly increase the biomass of the aboveground part of maize. Pot experiments further confirmed that strain BY2G20 could promote the growth of maize under high-salt conditions (Fig. 8E and F), but the effect was not apparent under salt-free conditions, which may be related to the ability of strain BY2G20 to adapt to a salt-alkaline environment.

### Conclusion.

This study provided a comprehensive understanding of a novel strain, BY2G20, isolated from saline-alkaline soil. Strain BY2G20 has typical PGPR characteristics and effectively promotes maize growth under high-salt conditions. The phylogenetic analysis of 16S rRNA and core genome sequences in combination with the ANI, AAI, and *in silico* DDH values confirmed that *M. dongyingensis* BY2G20 represents a novel species in the genus *Metabacillus*. Comparative genomic analysis of BY2G20 with closely related species showed high levels of evolutionary plasticity derived by HGTs, with the existence of strain-specific genes related to ISs, plasmids, prophages, and GIs, which facilitated adaptive evolution. Different degrees of purifying selection constrained the evolution of functional genes, which may have resulted in specialized processes such as salt tolerance and pH adaptability, enabling adaptation to saline-alkaline soil. *In vitro* experiments screened different plant growth-promoting traits such as salt and alkali tolerance, IAA production, and biocontrol activity in BY2G20. Systematic analysis of the BY2G20 genome identified the genetic basis that contributed to the adaptation to environmental niche, especially for genes related to salt tolerance, pH adaptability, and plant growth promotion. This study also demonstrated that inoculation of BY2G20 improved plant growth and assisted in the tolerance to NaCl stress. Therefore, crop plants inoculated with PGPRs could be a sustainable option for alleviating NaCl stress injury in seedlings. This study enhances our understanding of the evolution and genomic adaptation of the PGPR strain *M. dongyingensis* sp. nov. BY2G20 in saline-alkaline environments. The results can provide a theoretical reference for BY2G20 commercialization as a potential candidate for the organic agriculture biofertilizers in saline-alkaline areas.

## MATERIALS AND METHODS

### Determination of the general characteristics and growth-promoting traits of strain BY2G20.

Fresh and single colonies of strain BY2G20 on LB medium were used to test the colony morphology. The anti-salt and anti-alkali ability of strain BY2G20 were tested on LB medium with different concentrations of NaCl and different pH values, respectively. The optical density at 600 nm of the strain cultivation was measured by a BioPhotometer plus (Eppendorf, Germany). The morphological characteristics of strain BY2G20 cells were observed by a fluorescence microscope (Zeiss, Germany). The microbial microbiochemical identification tubes of Haibo Biological Technology Co., Ltd., were used to identify the carbon source utilization of strain BY2G20. The cellular fatty acid composition of strain BY2G20 was tested by the Agricultural Culture Collection of China (ACCC). The tests for IAA secretion and microorganism (Xanthomonas axonopodis, Ralstonia solanacearum, Fusarium *moniliforme*, Fusarium oxysporum, *Verticillium dahlia*, Escherichia coli DH5α, and Bacillus subtilis 168) antagonism were conducted following the methods formerly reported by our group (43).

### Genome sequencing and analysis.

A Bacteria Extraction kit (CWBIO Co., Ltd., China) was used for DNA extraction from isolates according to the manufacturer’s instructions. The whole genome of *M. dongyingensis* BY2G20 was sequenced using the HiSeq 4000-PE150 (Illumina) and PacBio RS II (Pacific Biosciences) platforms. The reads produced with the PacBio RS II were *de novo* assembled using MaSuRCA 2.2.1 (44). The genome sequence was annotated using the Rapid Annotations using Subsystems Technology (RAST) (45). The genome data were deposited in NCBI GenBank (accession number GCA_019933155.1).

The 16S rRNA sequence was compared with the taxonomically united 16S rRNA database in EzBioCloud (12) and LPSN (46) to identity similar species, whose 16S rRNA and genome sequences were then collected for subsequent analyses. The collected genomes were re-annotated using the RAST server (45). The ANI, AAI, and *in silico* DDH were calculated using the JSpecies 1.2.1 (47), CompareM (https://github.com/dparks1134/CompareM), and the genome-to genome distance calculator 2.1 (GGDC) (14), respectively. Two genomes that share >70% DDH and >95% ANI are typically considered to be members of the same species (14, 47).

### Comparative genomic analysis.

The features of the chromosomes and plasmids and comparisons thereof were performed using BLAST Ring Image Generator (BRIG) (48). The prophages were predicted using the PHAge search tool—Enhanced Release (PHASTER) (49). IslandViewer 4 was utilized to identify the genomic islands (50). Insertion sequences were predicted using ISFinder (51). We identified the functional category of the proteins using the COG assignment (52). The genes encoding carbohydrate binding and metabolic enzymes were identified using the dbCAN2 database by HMMER (53). The BY2G20 chromosome was analyzed for the presence of horizontal genes using HGTector (54). *Bacillus* (rank, genus; taxon ID 1386) and *Bacillaceae* (rank, family; taxon ID 186817) were set as self-group and close group, respectively. The horizontal genes among BY2G20 genomes and potential donors were identified and extracted from the HGTector output files. Syntenic analysis was achieved via ProgressiveMauve (55), which enabled the identification of locally colinear blocks (LCBs) using default parameters. For selection pressure analysis, ParaAT was used to codon-based align the orthologous genes between BY2G20 and *M. idriensis* DSM-19097^T^ (56), and the KaKs_Calculator 2.0 was used to compute the nonsynonymous (*K_a_*) and synonymous (*K_s_*) substitution rates (*K_a_*/*K_s_*) (57). The gene cluster related to secondary metabolism was identified and analyzed using antiSMASH (58) with the default parameters. The KEGG annotation (the KO functional orthologs for each pathway described by Zhang et al.) (40) in combination with local BLASTp searches with E value < 1E–10 and identity > 60% (the reference protein sequences for the IAA synthetic pathway in Bacillus velezensis SQR9 described by Shao et al. [41]) were performed to identity the Trp-dependent IAA synthesis-related genes in BY2G20.

### Phylogenetic analysis based on the core genome.

The collection consisted of the BY2G20 genome and 31 reference genomes from similar species (see Table S1). Orthologous groups of protein families were delimited using OrthoFinder software (59). The single-copy core gene families were extracted from the OrthoFinder output files. Nucleotide sequences of the single-copy core gene families were extracted according to the protein accession numbers and then aligned using MAFFT software (60). The core genome tree was constructed using the single nucleotide polymorphisms (SNPs) set present in single-copy core gene families. The SNPs set was integrated according to the arrangement of the genes on the BY2G20 chromosome. The putative recombinational regions were identified and removed from the SNPs set using ClonalFrameML software (61). The ML tree was constructed using MEGA 7 software (62) with the general time-reversible (GTR) model and 100 bootstrap replicates.

### Maize seedling growth analysis on nutrient solution.

Uniformly sized maize seeds were selected and sterilized. The maize seeds (dipped by strain BY2G20 in 10^6^ CFU/mL liquid or not) were germinated and grown on half-strength Hoagland’s nutrient solution supplemented with 100 mM NaCl or not. This experiment was divided into four groups: the group without strain BY2G20 and no 100 mM NaCl (BY2G20^−^ and NaCl^−^), the group with strain BY2G20 but no 100 mM NaCl (BY2G20^+^ and NaCl^−^), the group without strain BY2G20 but with 100 mM NaCl (BY2G20^−^ and NaCl^+^), and the group with strain BY2G20 and 100 mM NaCl (BY2G20^+^ and NaCl^+^). The method followed Lu’s report (63).

### Pot experiment.

Through this experiment, the effects of applying strain BY2G20 on the growth of maize seedlings under high NaCl conditions in the soil were tested. This experiment was divided into four groups: the group without strain BY2G20 and no 100 mM NaCl (BY2G20^−^ and NaCl^−^), the group with strain BY2G20 but no 100 mM NaCl (BY2G20^+^ and NaCl^−^), the group without strain BY2G20 but with 100 mM NaCl (BY2G20^−^ and NaCl^+^), and the group with strain BY2G20 and 100 mM NaCl (BY2G20^+^ and NaCl^+^). Uniformly sized maize seeds were selected and then sterilized for planting and then thinned when there were two leaves. Two-hundred milliliters of 100 mM NaCl was irrigated 2 days after thinning in the NaCl-treated groups, and the other two groups were irrigated with an equal amount of sterile water. Two days after NaCl treatment, a 200-mL solution containing 10^6^ CFU/mL of strain BY2G20 was used to irrigate the maize plants in the strain-treated groups, and the other two groups were irrigated with an equal amount of sterile water. The initial data were measured and recorded. During the experiment, the stem diameter and physiological plant height (the maximum height of the plant after straightening) were measured every 7 days. Seedlings were harvested on day 28 and their agronomic characteristics (including aboveground dry and fresh weights, belowground dry and fresh weights) were measured. This experiment was operated according to previously reported work by our group (64).
