# Supplementary figures and images for: Metabacillus dongyingensis sp. nov. Is Represented by the Plant Growth-Promoting Bacterium BY2G20 Isolated from Saline-Alkaline Soil and Enhances the Growth of Zea mays L. under Salt Stress
Source: mSystems. 2022 Mar 1;7(2):e01426-21. doi: 10.1128/msystems.01426-21 (PMC9040632; doi:10.1128/msystems.01426-21)

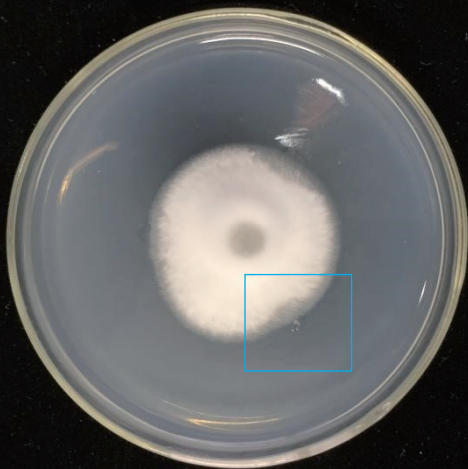

Supplement: FIG S1 [file msystems.01426-21-sf001.pdf]

A

Neighbour-joining tree

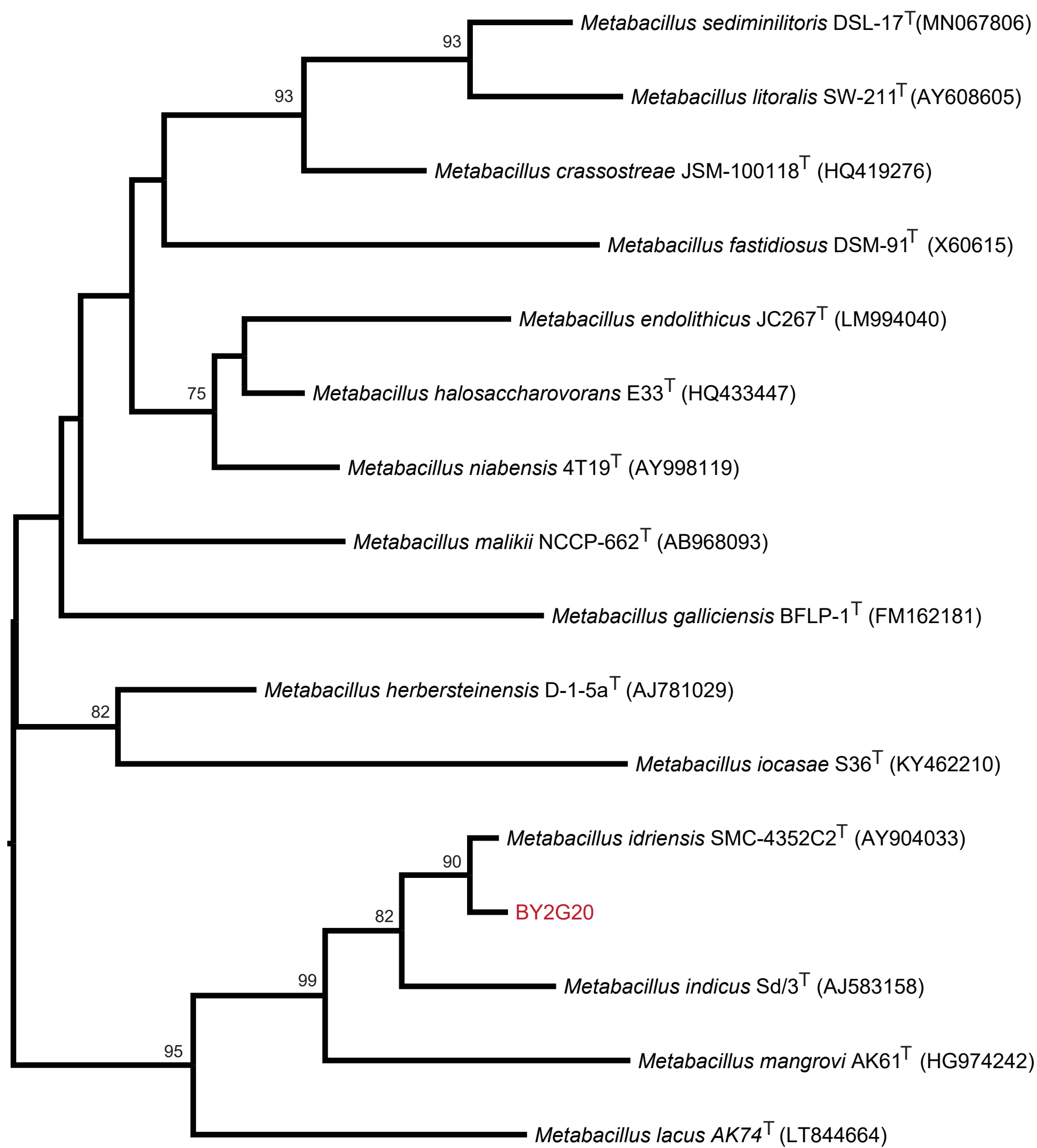

0.003

B

Minimum-evolution tree

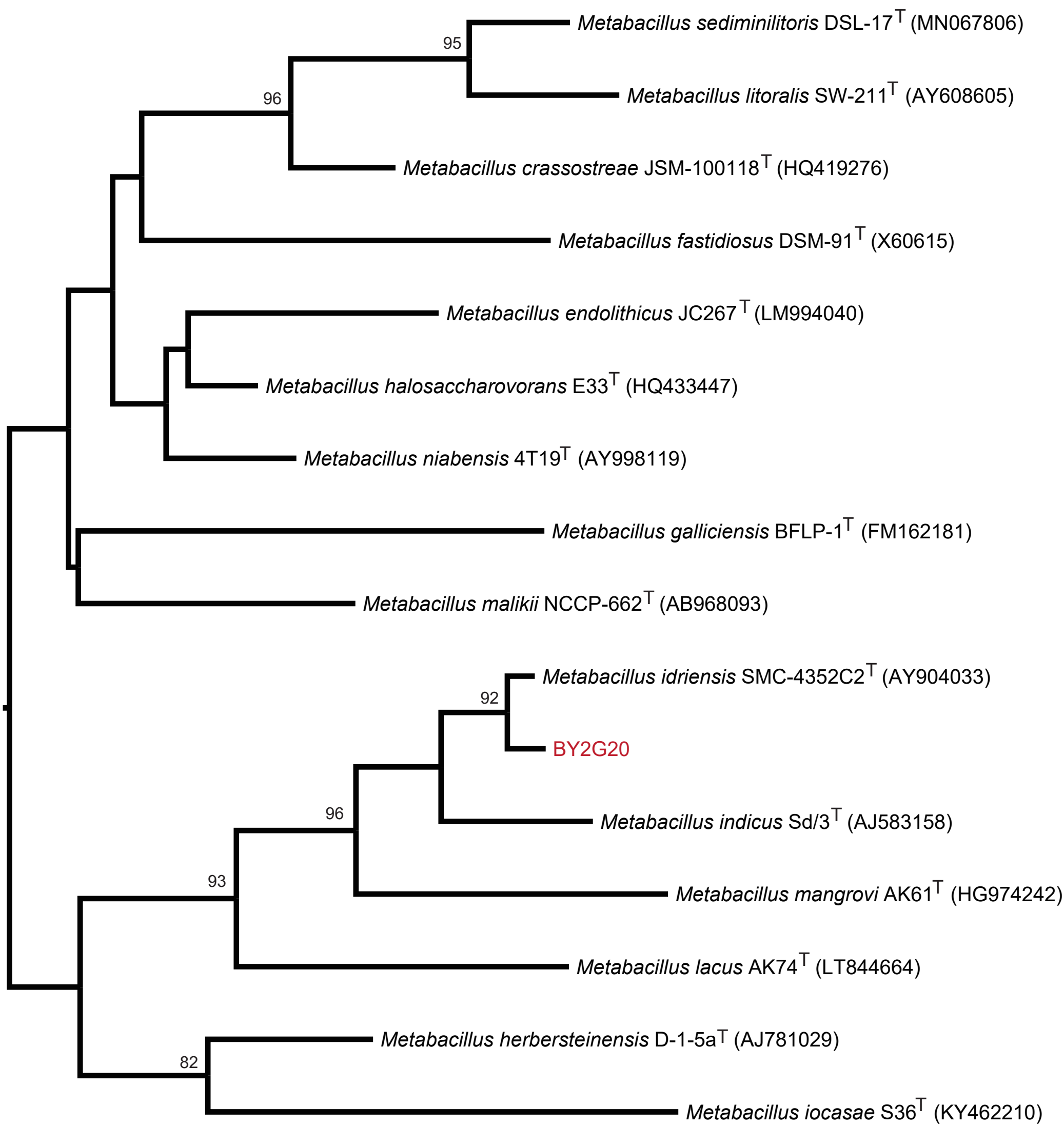

0.004

C

BGC2 (2668696 - 2692606)

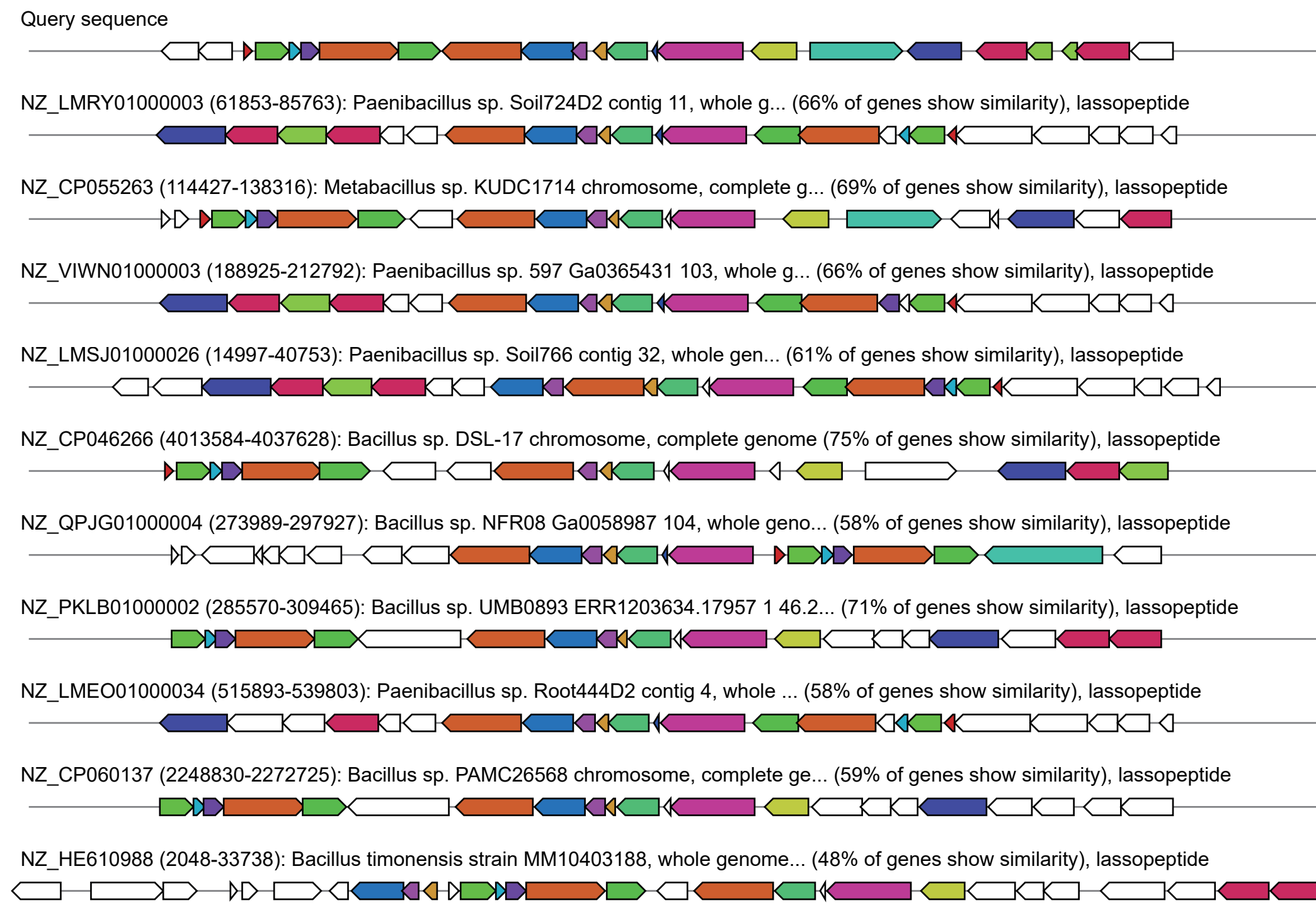

BGC 4 (2782566 - 2803706)

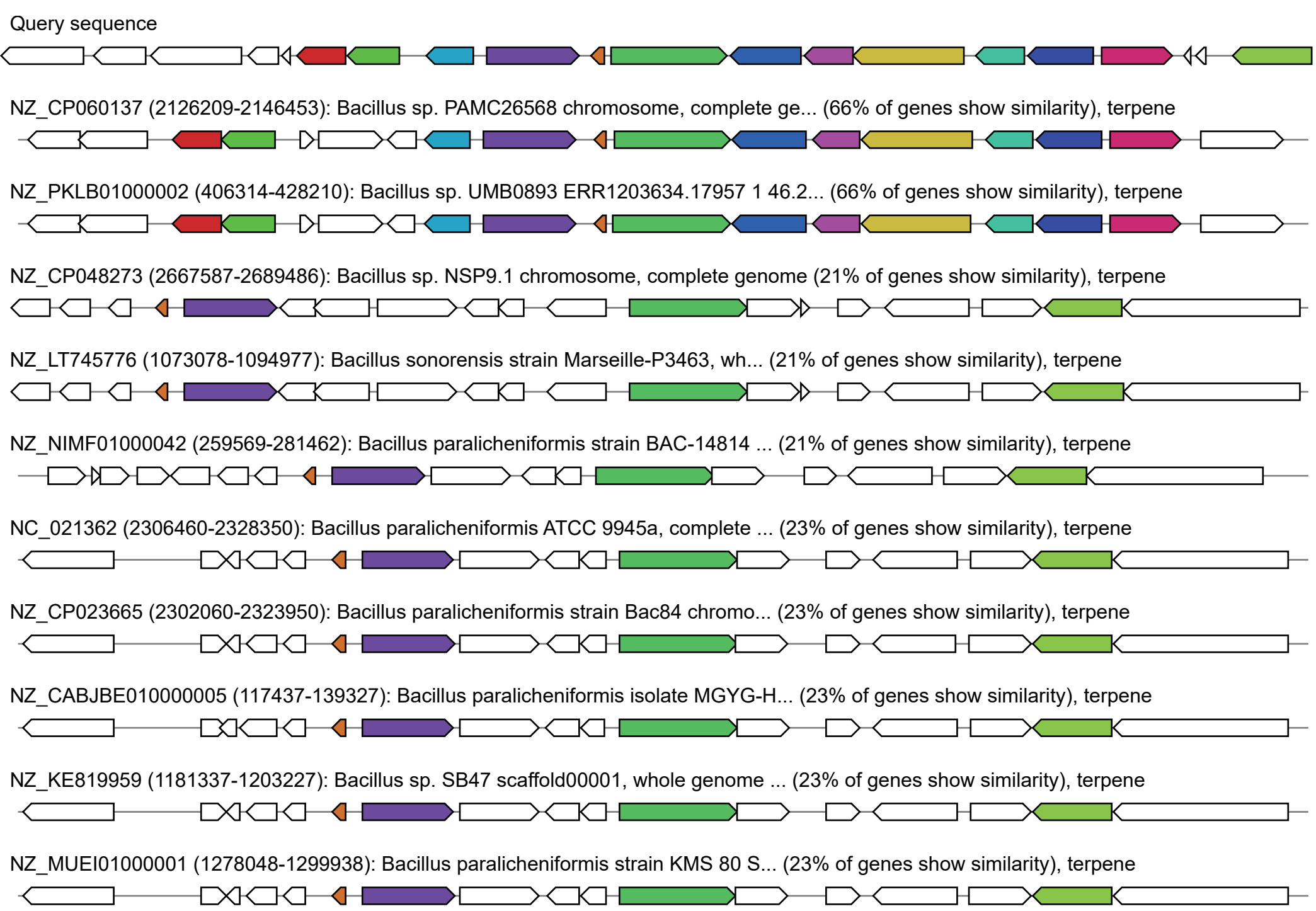

BGC 7 (3261363 - 3302463)

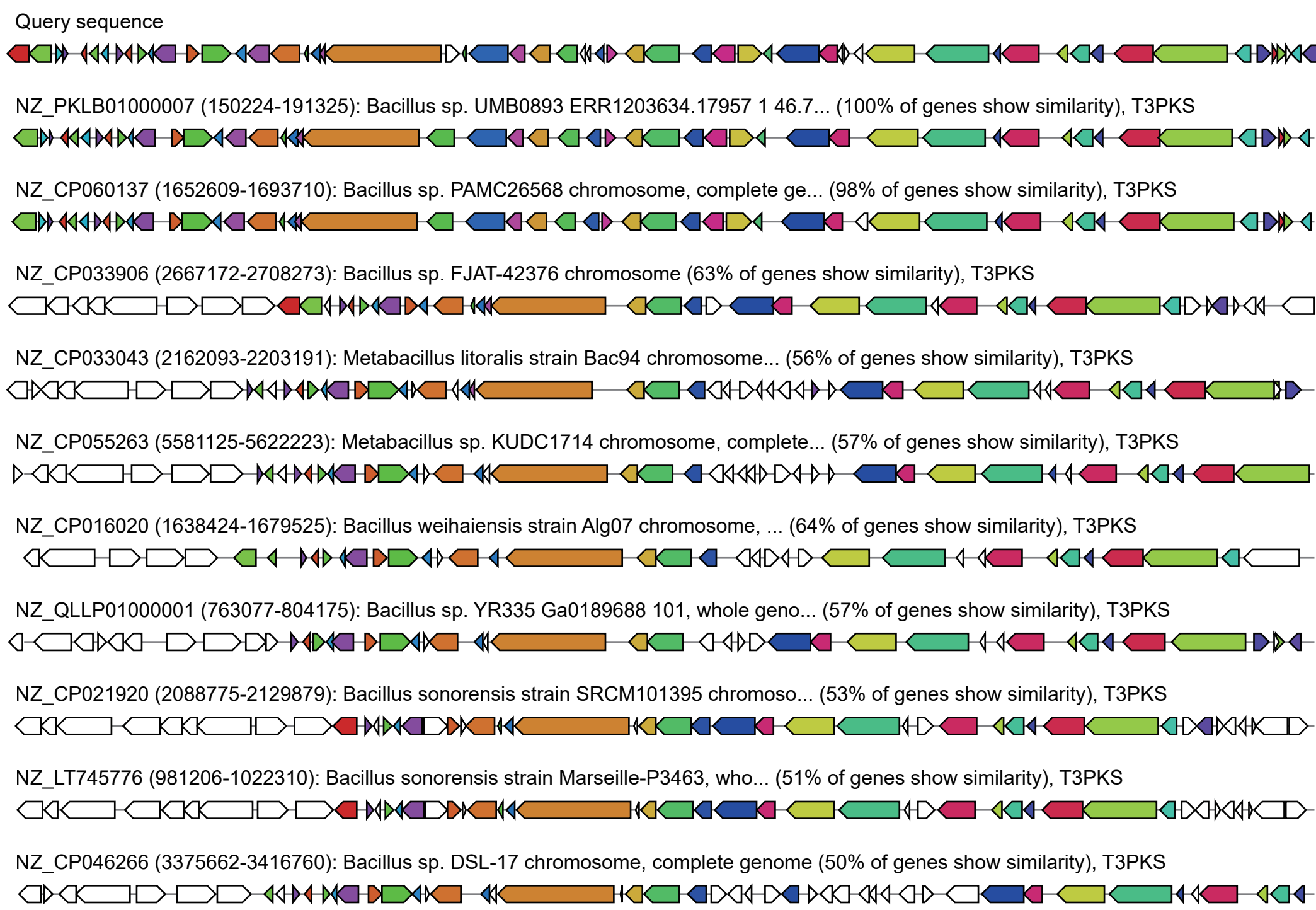

BGC 3 (2723881 - 2739317)

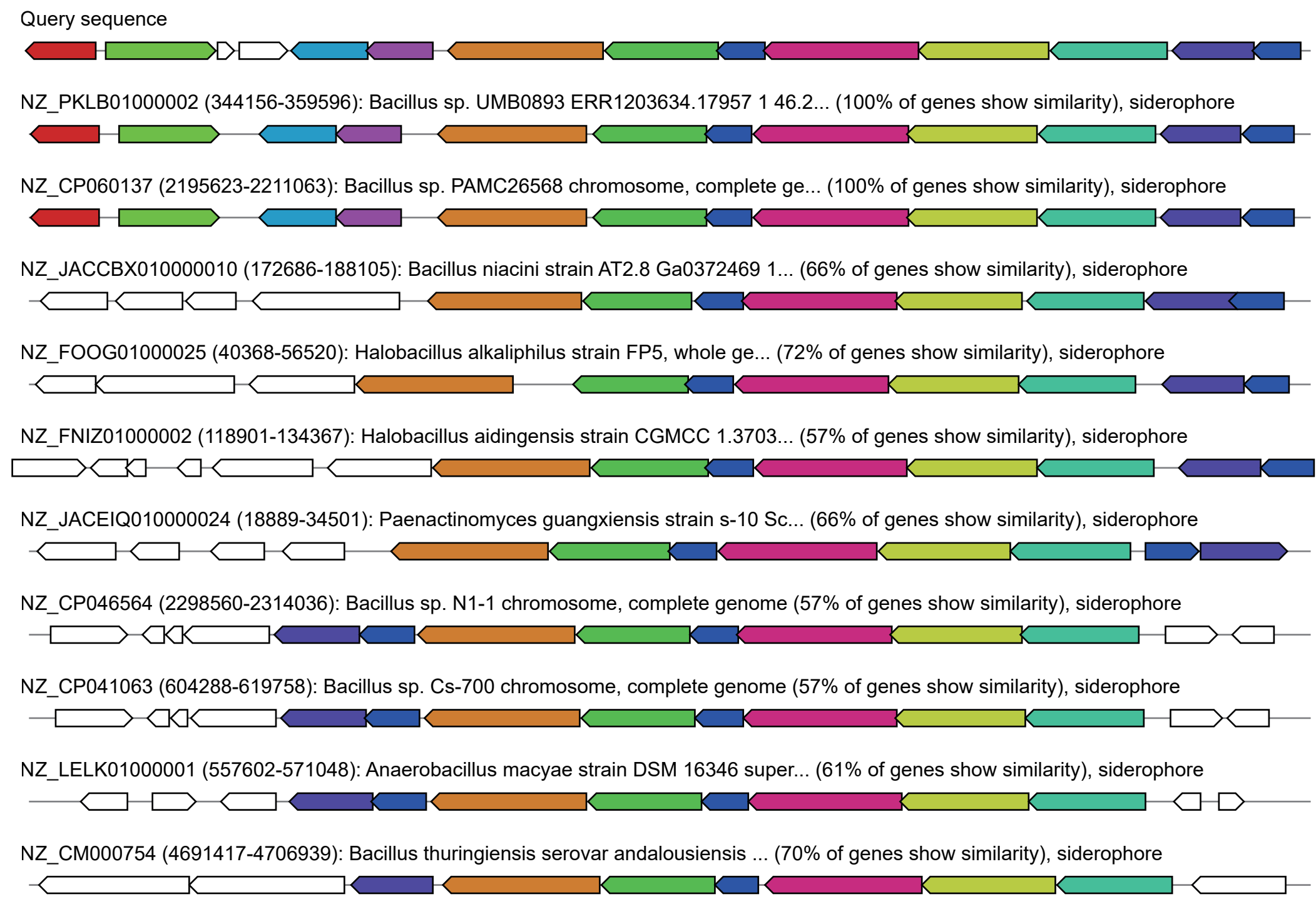

BGC 5 (2940637 - 3010533)

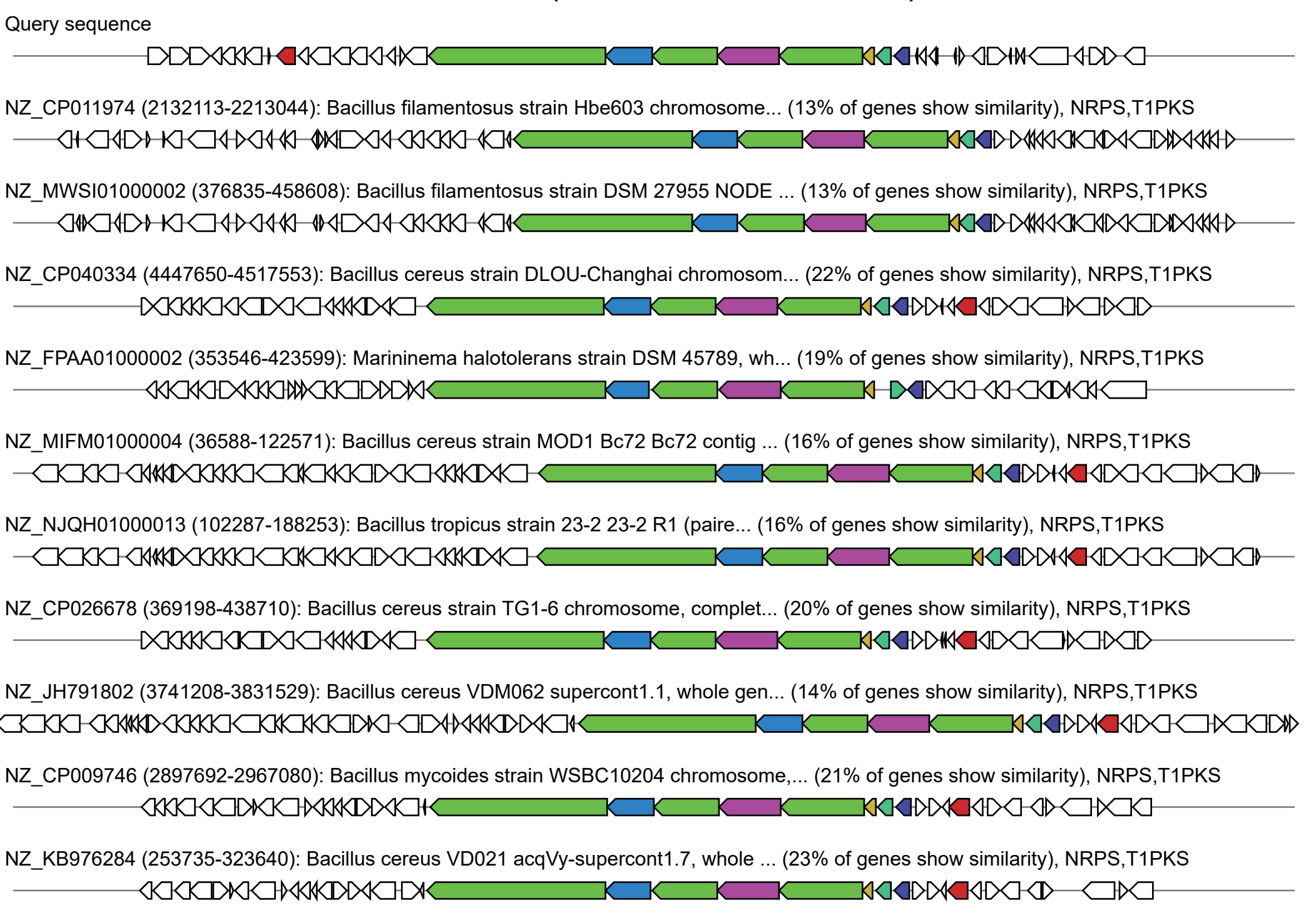

BGC 8 (4062489 - 4086025)

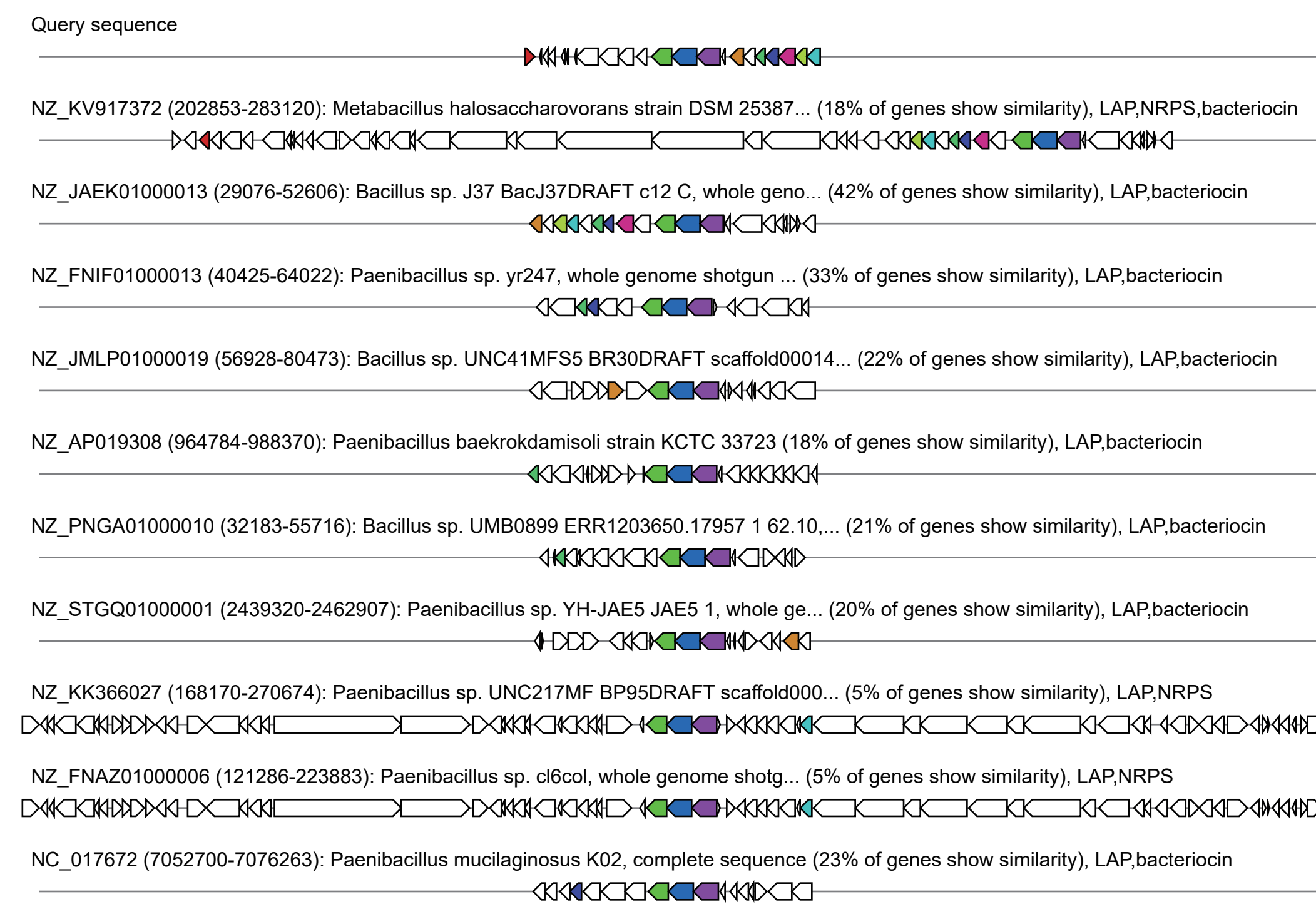

Supplement: FIG S2 [file msystems.01426-21-sf002.pdf]
