# Supplementary material for: Metabacillus dongyingensis sp. nov. Is Represented by the Plant Growth-Promoting Bacterium BY2G20 Isolated from Saline-Alkaline Soil and Enhances the Growth of Zea mays L. under Salt Stress
Source: mSystems. 2022 Mar 1;7(2):e01426-21. doi: 10.1128/msystems.01426-21 (PMC9040632; doi:10.1128/msystems.01426-21)
Supplement: TABLE S1 [file msystems.01426-21-st001.docx]

Table S1. Fatty acid composition of BY2G20 compared with the closely related type strains of the genus *Metabacillus*.

1. BY2G20; 2. *M. idriensis* DSM 19097^T^; 3. *M. indicus* MTCC 4374^T^; 4. *M. mangrovi* AK61^T^; 5. *M. lacus* AK74^T^; Results are presented as a percentage of the total fatty acids. Fatty acids amounting to 10% or more of the total fatty acids are in bold. ND, not detected. NA, no data. Summed feature 3 comprised C_16:1_ ω7c / C_16:1_ ω6c; summed feature 4 comprised iso-C_17:1_ I / anteiso-C_17:1_ B; summed feature 5 comprised anteiso-C_18:0_ / C_18:2_ ω6,9c; summed feature 8 comprised C_18:1_ ω7c / C_18:2_ ω6c.

| **Fatty acid composition** | 1 | 2 | 3 | 4 | 5 |
| --- | --- | --- | --- | --- | --- |
| C_12:0_ | 0.1 | NA | NA | NA | NA |
| iso-C_13:0_ | 0.1 | ND | ND | ND | 2.7 |
| anteiso -C_13:0_ | 0.2 | NA | NA | NA | NA |
| C_14:0_ | 1.1 | ND | 6.8 | 1.3 | 3.8 |
| iso-C_14:0_ | 5.5 | **24.9** | 6.0 | **15.9** | 1.1 |
| iso-C_15:0_ | **16.7** | **15.2** | **24.0** | **23.8** | **11.8** |
| anteiso-C_15:0_ | **30.0** | **30.3** | **14.8** | **40.4** | **10.1** |
| C_16:0_ | **17.7** | 2.2 | **13.5** | 2.6 | **14.1** |
| iso-C_16:0_ | 8.0 | **14.6** | 9.2 | 7.2 | 3.6 |
| C_16:0_ 2OH | 0.1 | ND | ND | ND | 2.2 |
| C_16:0_ 3OH | 0.1 | NA | NA | NA | NA |
| C_16:1_ ω11c | 3.7 | 1.6 | 8.6 | ND | 5.4 |
| C_16:1_ ω5c | 0.1 | NA | NA | NA | NA |
| C_16:1_ ω7c alcohol | 0.7 | 5.4 | 4.9 | 1.1 | 5.3 |
| C_17:0_ | 0.4 | ND | ND | ND | 3.2 |
| iso-C_17:0_ | 4.5 | 1.6 | ND | <1 | 5.2 |
| anteiso-C_17:0_ | 6.8 | 4.3 | 5.1 | 4.0 | 9.6 |
| C_17:0_ cyclo | 0.1 | NA | NA | NA | NA |
| C_17:1_ iso ω10c | 0.6 | NA | NA | NA | NA |
| C_18:0_ | 0.9 | ND | ND | <1 | 1.6 |
| iso-C_18:0_ | 0.2 | NA | NA | NA | NA |
| C_18:0_ 3OH | 0.1 | NA | NA | NA | NA |
| C_18:1_ ω9c | 0.4 | NA | NA | NA | NA |
| iso-C_19:0_ | 0.1 | NA | NA | NA | NA |
| anteiso-C_19:0_ | 0.1 | NA | NA | NA | NA |
| C_20.0_ | 0.1 | NA | NA | NA | NA |
| iso-C_20:0_ | 0.1 | NA | NA | NA | NA |
| Summed Feature 3 | 0.4 | ND | ND | ND | 1.4 |
| Summed Feature 4 | 0.5 | ND | ND | ND | 5.6 |
| Summed Feature 5 | 0.5 | NA | NA | NA | NA |
| Summed Feature 8 | 0.3 | NA | NA | NA | NA |
